# Supplementary material for: Liver and pancreatic-targeted interleukin-22 as a therapeutic for metabolic dysfunction-associated steatohepatitis
Source: Nat Commun. 2024 May 29;15:4528. doi: 10.1038/s41467-024-48317-x (PMC11137118; doi:10.1038/s41467-024-48317-x)
Supplement: Supplementary file 1 — Supplementary Information [file 41467_2024_48317_MOESM1_ESM.pdf]

Liver and Pancreatic-Targeted Interleukin-22 as a Therapeutic for Metabolic Dysfunction-Associated Steatohepatitis

Haressh Sajiir[1,2#], Sahar Keshvari[1,2#], Kuan Yau Wong[1,2], Danielle J. Borg[1,2], Frederik J. Steyn[2], Christian Fercher[3,4], Karin Taylor[4], Breten Taylor[4], Ross T. Barnard[3,4], Alexandra Mueller[1,2], Md Moniruzzaman[1,2], Gregory Miller[2,5], Ran Wang[1,2], Amelia Fotheringham[1,2], Veronika Schreiber[1,2], Yong Hua Sheng[1,2], Janelle Louise Hancock[6], Dorothy Loo[6], Lucy Burr[1,2,7] , Tony Huynh[8,9,10,11], Jack Lockett[1,2,11], Grant A. Ramm[2, 12], Graeme A. Macdonald[2, 13], Johannes B. Prins[14], Michael A. McGuckin[15], Sumaira Z. Hasnain[1,2,16*]

[1]Immunopathology Group, Mater Research Institute-The University of Queensland, Translational Research Institute, Brisbane, Australia

[2] Faculty of Medicine, The University of Queensland, Brisbane, QLD, Australia

[3] Australian Research Council Training Centre for Biopharmaceutical Innovation, Australian Institute for Bioengineering and Nanotechnology, The University of Queensland, Brisbane, Australia

[4] School of Chemistry and Molecular Biosciences, Faculty of Science, The University of Queensland, Brisbane, QLD, Australia

[5] Envoi Specialist Pathologists, Kelvin Grove, Brisbane, Australia

[6] Proteomics Core Facility, Translational Research Institute, Brisbane, Australia

[7] Department of Respiratory and Sleep Medicine, Mater Health, South Brisbane, Australia.

[8]Department of Endocrinology & Diabetes, Queensland Children's Hospital, South Brisbane, Queensland, Australia

[9]Children's Health Research Centre, Faculty of Medicine, The University of Queensland, Brisbane, Australia.

[10]Department of Chemical Pathology, Mater Pathology, South Brisbane, Queensland, Australia.

[11]Department of Diabetes and Endocrinology, Princess Alexandra Hospital, Brisbane, Queensland, Australia

[12] Hepatic Fibrosis Group, QIMR Berghofer Medical Research Institute, Brisbane, Queensland, Australia

[13] Department of Gastroenterology and Hepatology, Princess Alexandra Hospital, Brisbane, Queensland, Australia.

[14] Health Translation Queensland, Royal Brisbane and Women's Hospital, Herston, Australia.

[15] Faculty of Medicine, Dentistry and Health Sciences, University of Melbourne, Victoria, Australia

[16] Australian Infectious Disease Research Centre, University of Queensland, Brisbane, Australia

[#] These authors contributed equally to the work
*Correspondence should be addressed to: Associate Professor Sumaira Z. Hasnain
Immunopathology Group, Mater Research Institute – The University of Queensland, Translational Research Institute, 37 Kent St, Woolloongabba, Brisbane, QLD 4102, Australia.
t: +61-7-34436939, f: +61-7-31632550; E: sumaira.hasnain@mater.uq.edu.au

Supplementary Information

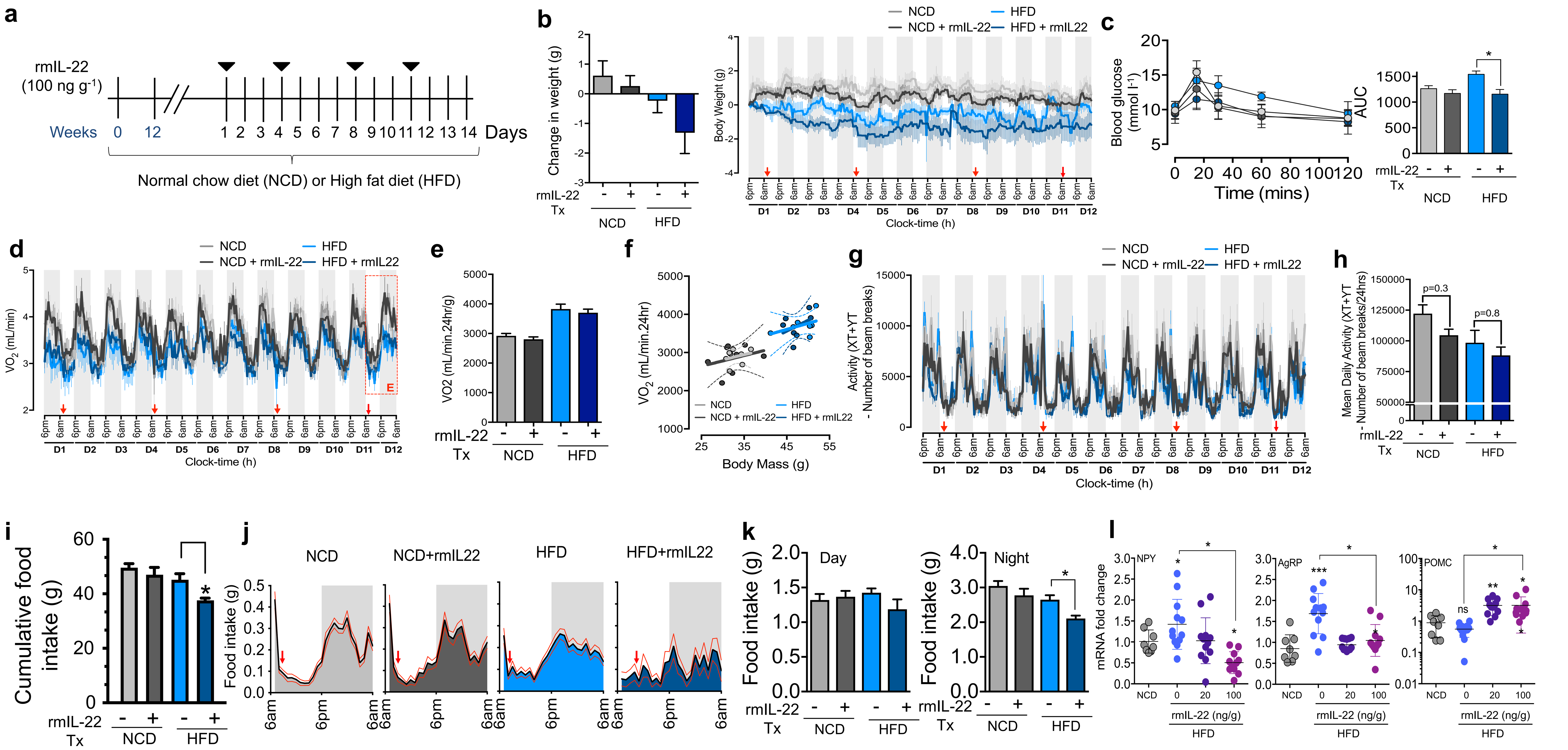

Supplementary Figure 1: **Recombinant mouse IL-22 improved glucose tolerance without altering metabolic rate or energy expenditure and may influence satiety.** (**a**) Schematic showing mice were fed a normal chow diet (NCD) or a high fat diet (HFD) for 14 weeks and treated biweekly for the last two weeks with 100 ng g^{-1} of recombinant mouse IL-22 (rmIL-22), i.p. Control NCD and HFD animals received PBS. (**b**) decrease in body weight of HFD animals treated with rmIL-22, no changes in NCD animals treated with rmIL-22. Average real-time measurements of body weight across the first 12 days of treatment recorded using TSE phenomaster system. Red arrows depict the rmIL-22 treatments. (**c**) Oral glucose tolerance test (oGTT) and area under the curve (AUC) shows significant improvements in hyperglycaemia with rmIL-22 treatment. No alterations in the (**d**) average VO_2 measurements (mL min^{-1}), (**e**) VO_2 on day 11, (**f**) or VO_2 when compared to body mass of the animals; ANCOVA was used to adjust for the influence of body weight. (**g**) Average real time activity and (**h**) activity of animals in the last 24 h as determined through beam breaks shows no differences between treated and untreated animals. (**i**) Change in cumulative food intake over the experimental period demonstrates the decrease in total food consumption in HFD animals treated with rmIL-22. Not seen in NCD animals treated with rmIL-22. (**j**) Food intake measurements on day 11 (average block colours, +/- SD in red) shows the reduced intake of HFD animals treated with rmIL-22 (**k**) shown that changes in food intake occur during the night only in the HFD animals treated with rmIL-22. (**l**) Gene expression changes in neuronal subpopulations. a-k: n=7-8; m: n = 8-12 ANOVA, Bonferroni's *post hoc* test. *p<0.05; ***p<0.001. Source data are provided as a Source Data file

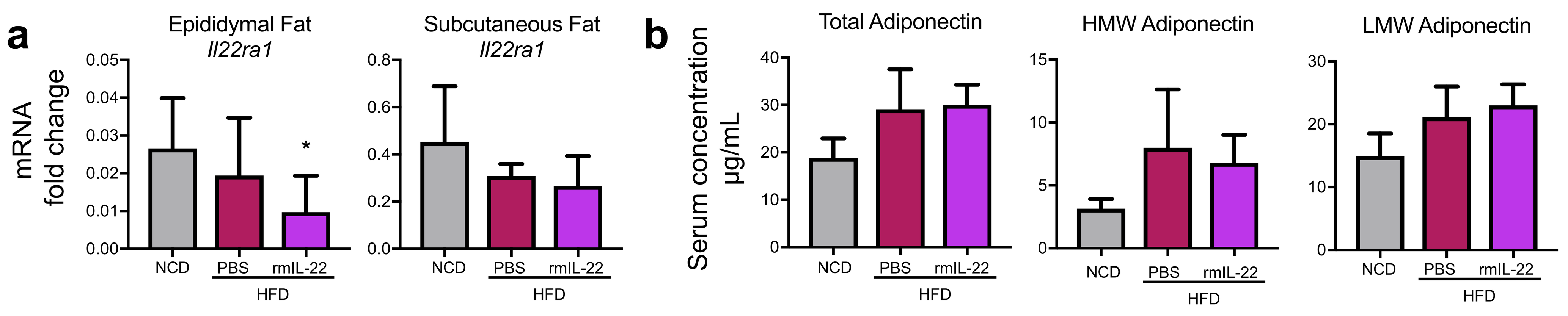

Supplementary Figure 2: **No change in adiponectin with rmIL-22 treatment.** Mice were fed a normal chow diet (NCD) or a high fat diet (HFD) for 14 weeks and treated biweekly for the last two weeks with 100 ng g^{-1} of recombinant mouse IL-22 (rmIL-22), i.p. Control NCD and HFD animals received PBS. Experimental schematic shown in Figure 1a. mRNA expression analyses for (**a**) IL-22 receptor (*Il22ra1*) (**b**) No significant changes were observed in the circulating serum levels of total, high molecular weight (HMW) or low molecular weight (LMW) adiponectin protein. n=8, ANOVA, Bonferroni's *post hoc* test. *p<0.05 compared to NCD mice. Source data are provided as a Source Data file

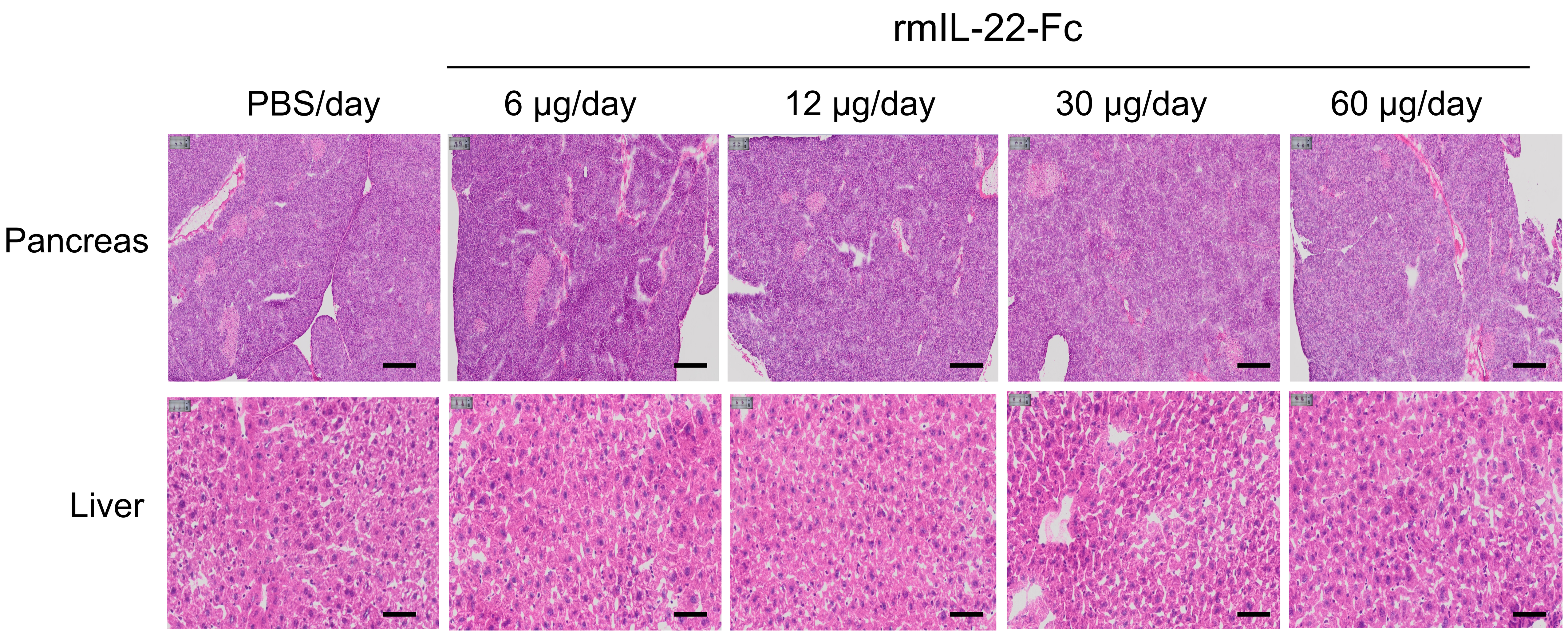

Supplementary Figure 3: **No major changes in pancreas and liver with chronic administration of long-circulating rmIL-22-Fc.** C57BL/6 animals were treated with either PBS or 6, 12, 30 or 60 µg of rmIL-22-Fc daily for 8 weeks (s.c.). Schematic shown in Figure 1f. H&E staining of pancreas (scale bar: 100 µm) and liver (scale bar: 50 µm).

a

hIL-22

MGWSCIILFLVATATGVHSLEEQKLISEEDLNAPISSHCRLDKSNFQQPYITNRTFMLAKEASLADNNTDVRLIGEKLFHGVSMSERCYLMKQVLNFTLEEVLFPQSDRFQPYMQEVVPFL
ARLSNRLSTCHIEGDDLHIQRNVQKLKDTVKKLGESGEIKAIGELDLLFMSLRNACIHHHHHH-

ScFv-hIL-22

MGWSCIILFLVATATGVHSELEVQLLESGGGLVQPGGSLRLSCAASGFTFSSYAMSWVRQAPGKGLEWVSSITAEGTHTWYADSVKGRFTISRDNSKNTLYLQMNSLRAEDTAVYYCAK
TSYRFDYWGQGTLVTVS**SGGGGSGGGGSGGGGS**DIQMTQSPSSLSASVGDRVTITCRASQSISSYLNWYQQKPGKAPKLLIYKASRLQSGVPSRFSGSGSGTDFTLTISSLQPEDFAT
YYCQQKWDPPRTFGQGTKVEIKRSGGGGSAPISSHCRLDKSNFQQPYITNRTFMLAKEASLADNNTDVRLIGEKLFHGVSMSERCYLMKQVLNFTLEEVLFPQSDRFQPYMQEVVPFL
ARLSNRLSTCHIEGDDLHIQRNVQKLKDTVKKLGESGEIKAIGELDLLFMSLRNACIEQKLISEEDLNHHHHHH-

hIL-22-ScFv

MGWSCIILFLVATATGVHSLEEQKLISEEDLNAPISSHCRLDKSNFQQPYITNRTFMLAKEASLADNNTDVRLIGEKLFHGVSMSERCYLMKQVLNFTLEEVLFPQSDRFQPYMQEVVPFL
ARLSNRLSTCHIEGDDLHIQRNVQKLKDTVKKLGESGEIKAIGELDLLFMSLRNACISGGGGSEVQLLESGGGLVQPGGSLRLSCAASGFTFSSYAMSWVRQAPGKGLEWVSSITAEGT
HTWYADSVKGRFTISRDNSKNTLYLQMNSLRAEDTAVYYCAKTSYRFDYWGQGTLVTVS**SGGGGSGGGGSGGGGS**DIQMTQSPSSLSASVGDRVTITCRASQSISSYLNWYQQKPG
KAPKLLIYKASRLQSGVPSRFSGSGSGTDFTLTISSLQPEDFATYYCQQKWDPPRTFGQGTKVEIKRHHHHHH-

hIL-22-GLP1

MGWSCIILFLVATATGVHSHGEGTFTSDLSKQMEEEAVRLFIEWLKNGGPSSGAPPPSGGSSSGGGSSSGGGSSSGGGSSSGAPISSHCRLDKSNFQQPYITNRTFMLAKEASLADNN
TDVRLIGEKLFHGVSMSERCYLMKQVLNFTLEEVLFPQSDRFQPYMQEVVPFLARLSNRLSTCHIEGDDLHIQRNVQKLKDTVKKLGESGEIKAIGELDLLFMSLRNACIGGSHHHHHHH
GGSEQKLISEEDL-

Key: Leader sequence; ScFv; human IL-22; GLP1 (Exe-4)

b

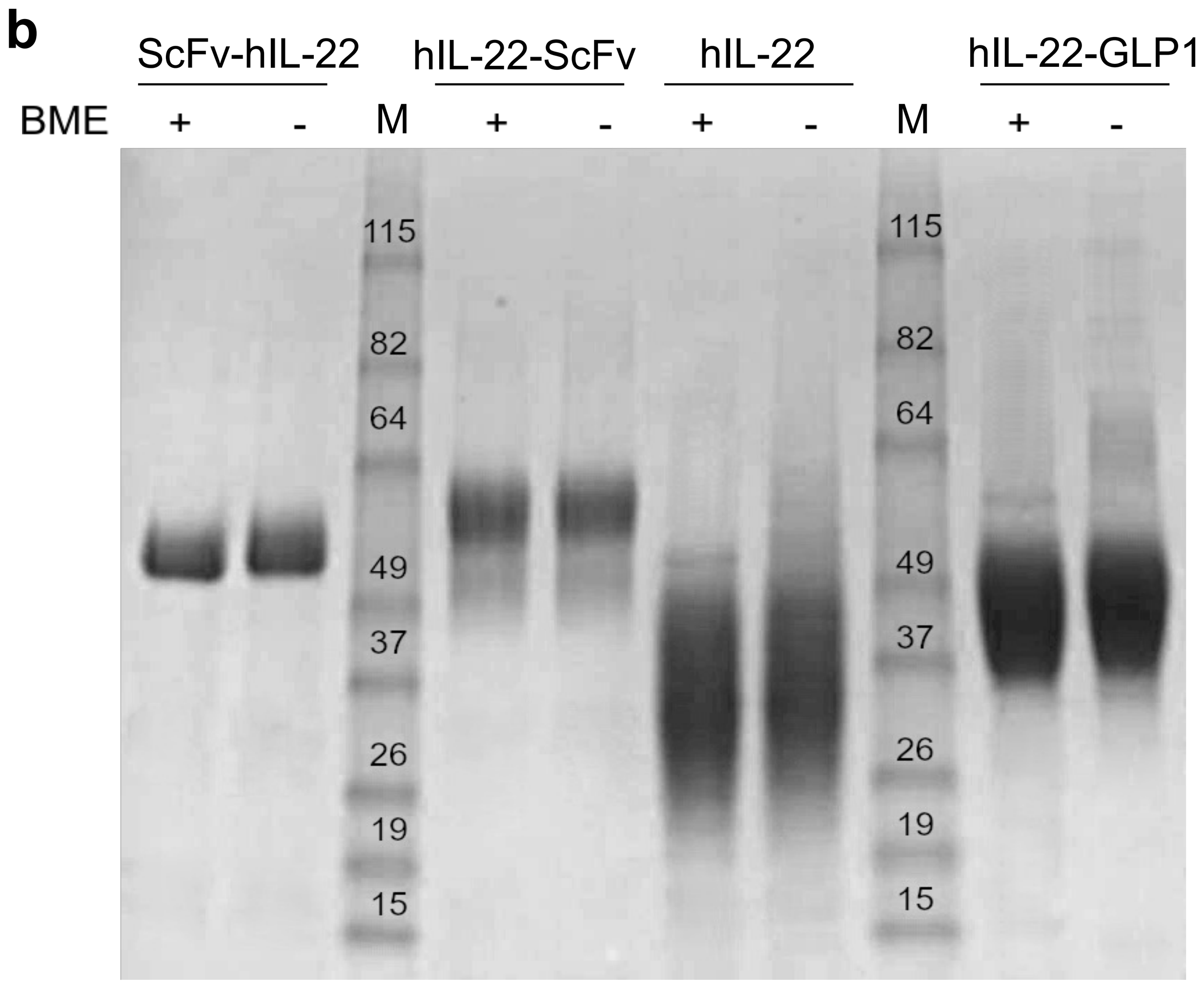

c

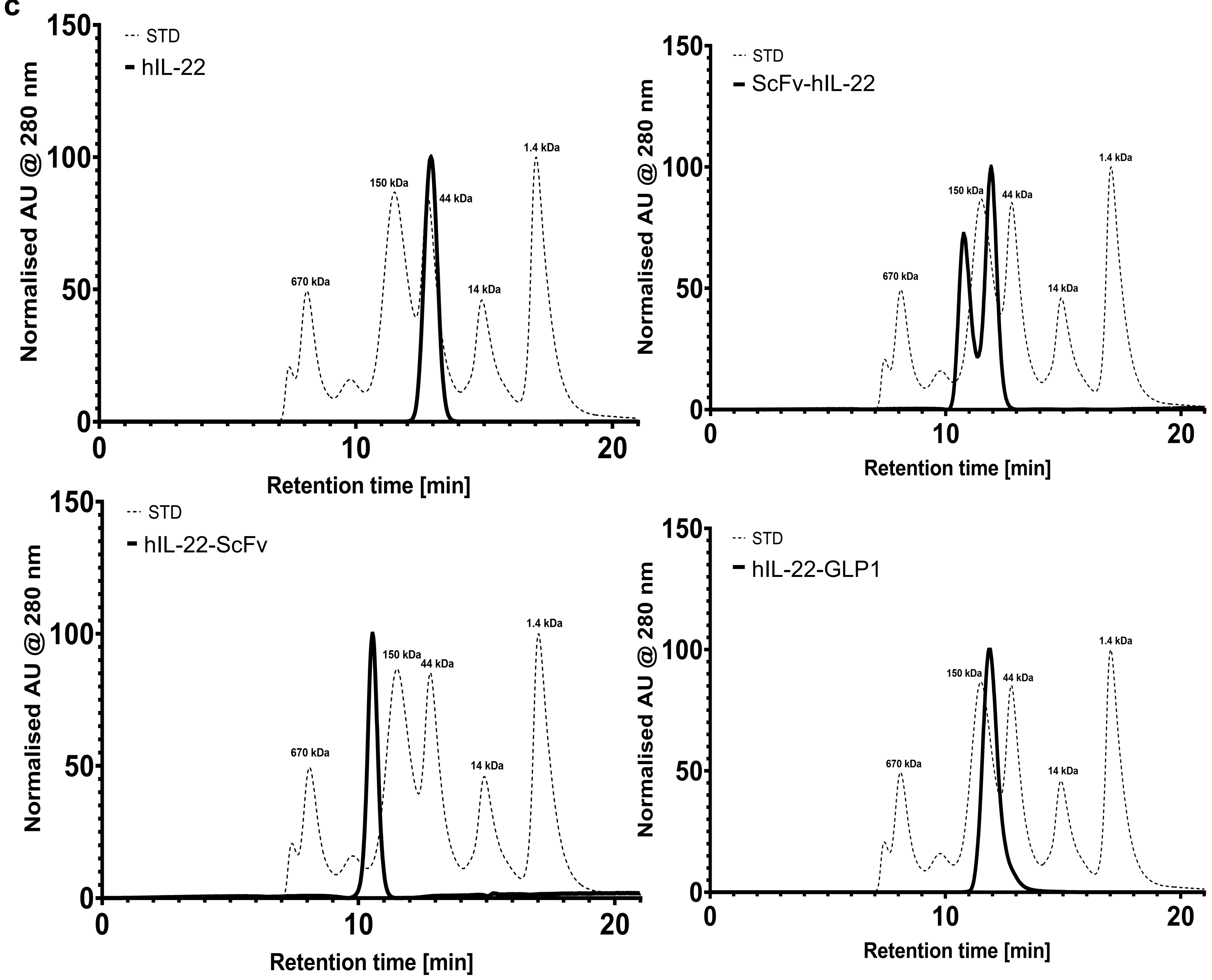

d

Clone	Apparent Mw [kDa]	Oligomer
hIL-22	37	2
ScFv-hIL-22	162 / 73	3 / 1
hIL-22-ScFv	187	4
hIL-22-GLP1	76	2

e

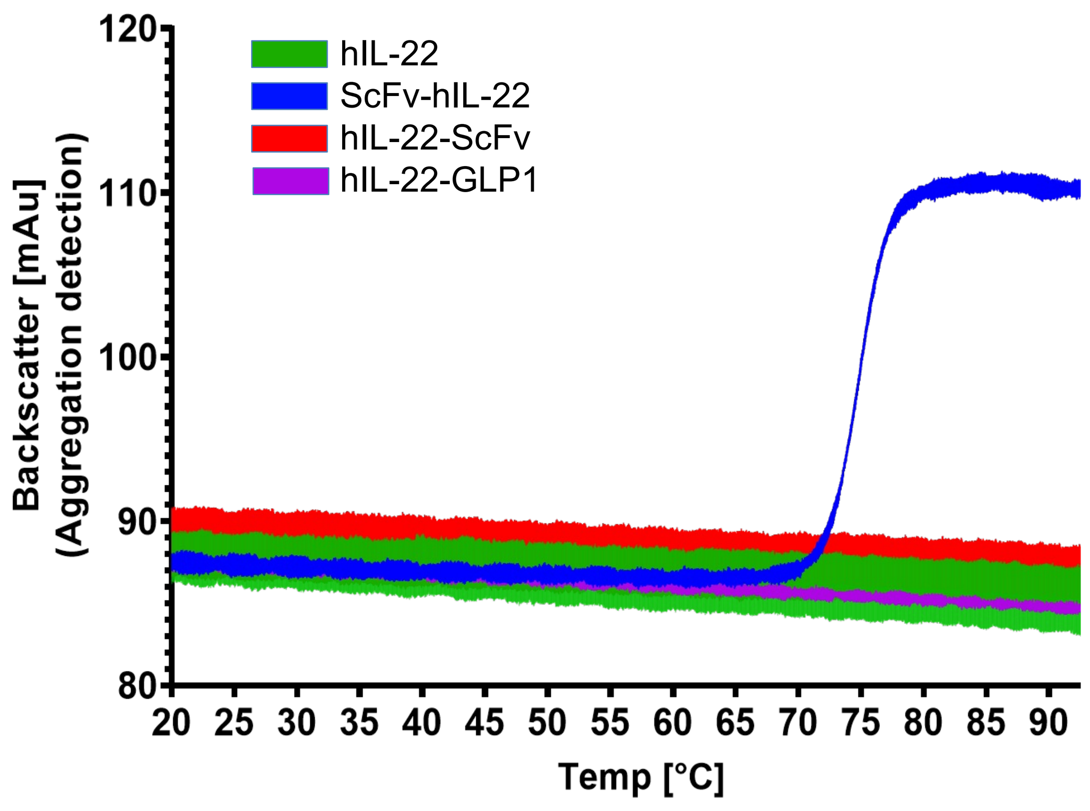

f

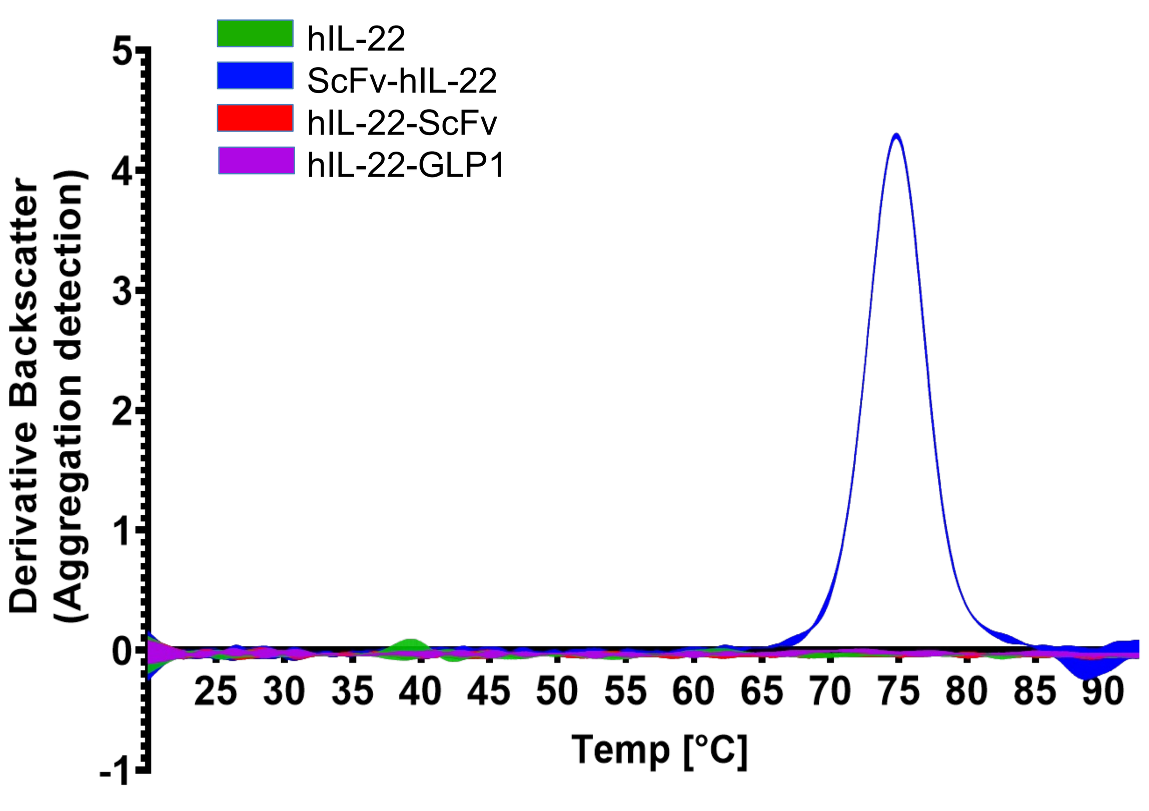

Supplementary Figure 4: Human IL-22-fusion proteins. (**a**) All proteins contained N-terminus Myc and a C-terminus His-tag. hIL-22-fusions contained linkers as shown in sequences above. (**b**) Purified proteins were analysed using western blotting under reducing (+BME) and non-reducing conditions (-BME). Lane 'M' shows the marker in kDa. (**c**) Analytical HPLC SEC aggregation analyses of purified proteins shows potential aggregation of ScFv-hIL-22. The apparent molecular weight (Mw) is shown in (**d**). (**e**) Aggregation and (f) aggregation derivative shows that ScFv-hIL-22 aggregates at higher temperatures and therefore may have low stability in-vivo. Source data are provided as a Source Data file

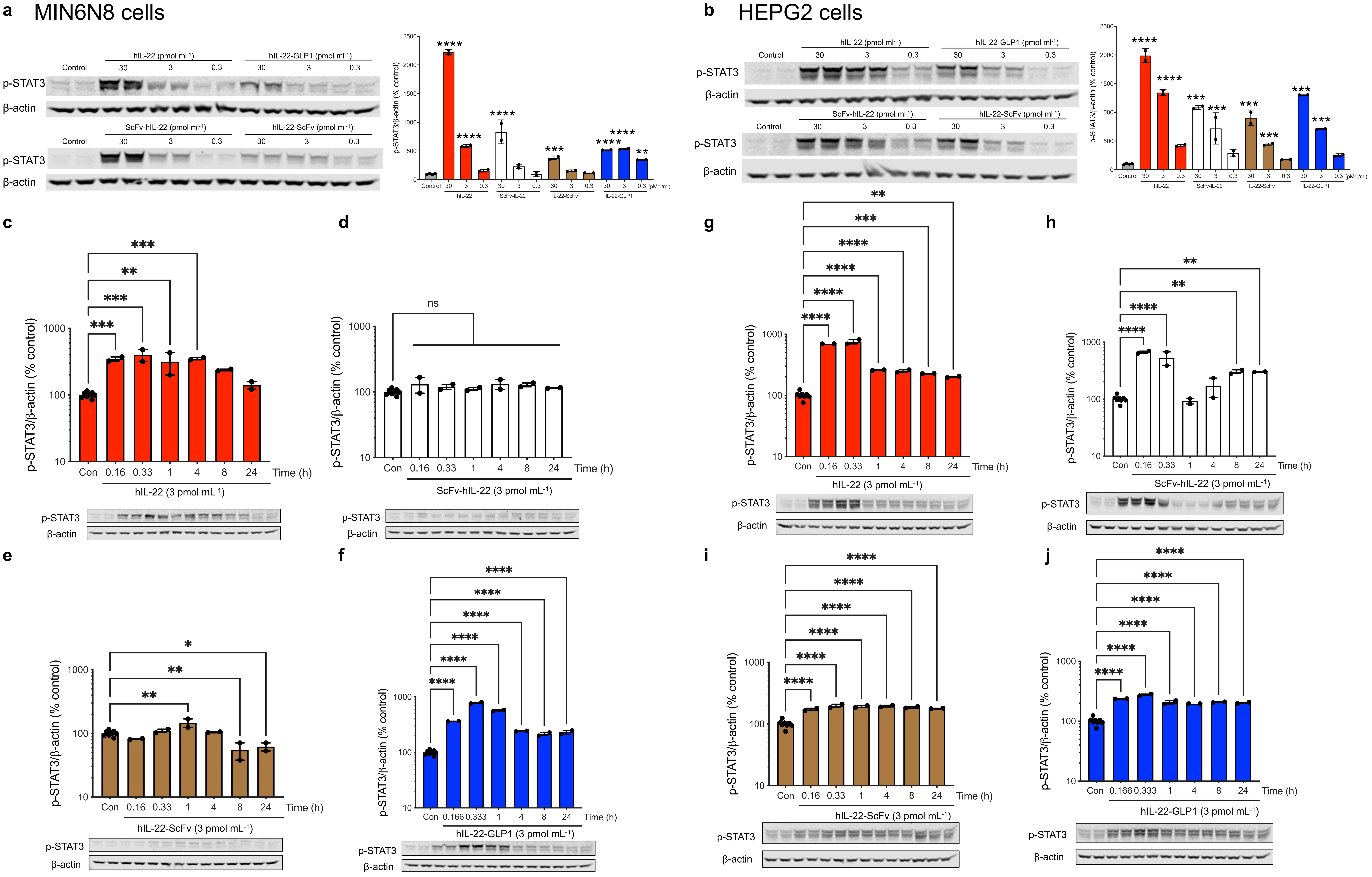

Supplementary Figure 5: Human IL-22-fusion proteins activate STAT3p in MIN6N8 and HEPG2 cell lines. (**a**) MIN6N8 and (**b**) HEPG2 cells were treated with hIL-22, ScFv-hIL-22, hIL-22-ScFv and hIL-22-GLP1 at 30, 3 and 0.3 pmol mL^{-1} concentration for 30 mins, protein was isolated and analysed using western blotting for STAT3-phophorylation. (**c-f**) MIN6N8 cells and (**g-j**) HEPG2 cells were stimulated with native hIL-22 and hIL-22 fusions were used at 3 pmol mL^{-1} concentration and STAT3p was assessed at 6 different time points (0.16, 0.33, 1, 4, 8, 24 h) to assess the kinetics of activation. Representative data from 2 independent experiments. PBS was used a vehicle control (con) for all experiments. ANOVA, Bonferroni's post hoc test. *p<0.05, **p<0.01, ***p<0.001, ****p<0.0001 compared to control. Source data are provided as a Source Data file

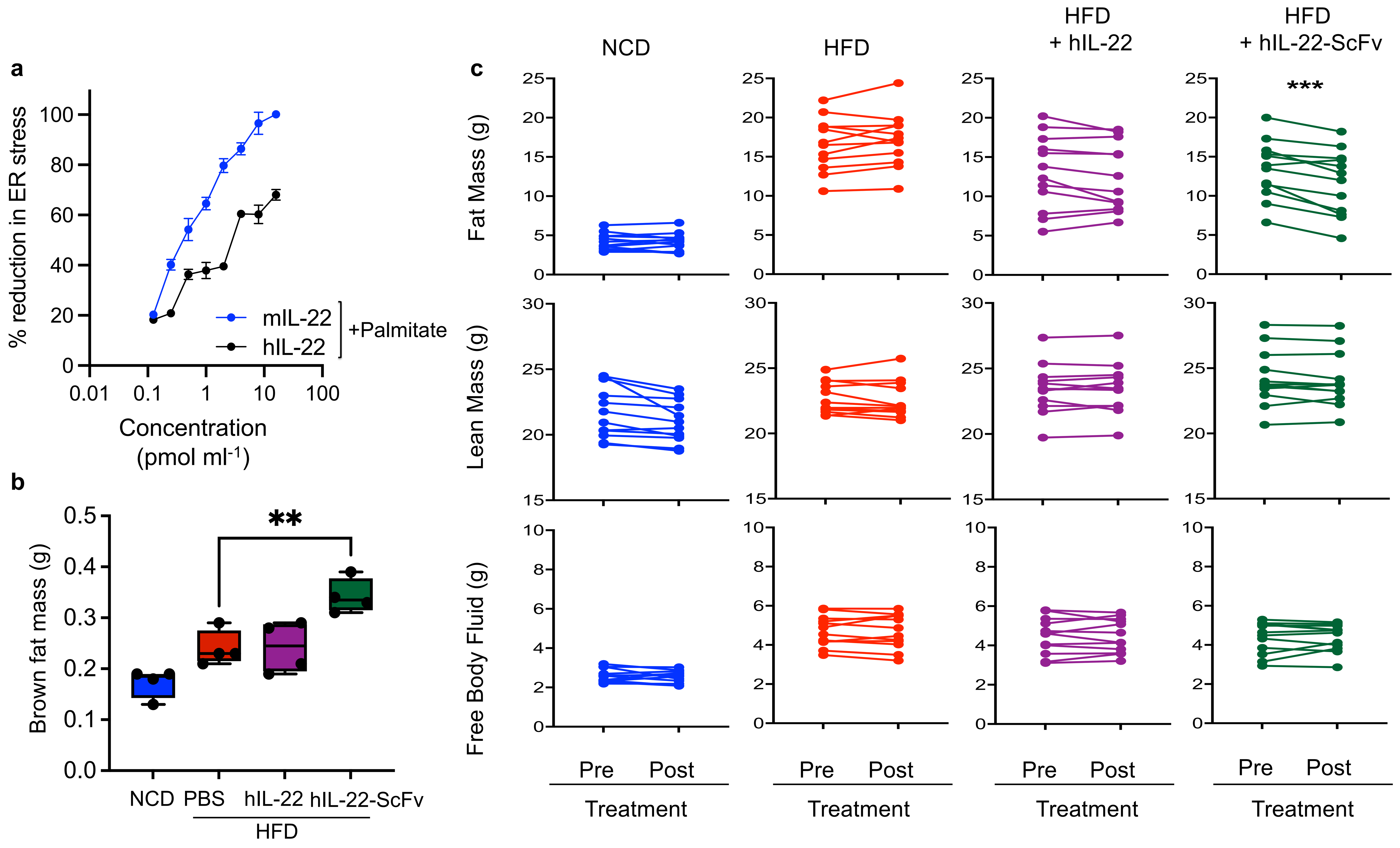

Supplementary Figure 6: Efficacy of hIL-22 and targeted hIL-22. (a) MIN6N8 cells transfected with the ERAI reporter were treated with 0.2 mM palmitate for 24 h prior to treatment with increasing doses of recombinant mouse IL-22 (mIL-22) or human IL-22 (hIL-22) at increasing concentrations (0.125 – 16 pmol mL^{-1}). Data presented as the percentage reduction from the palmitate only. n = 4. (**b**) Brown fat (**c**) and Nuclear magnetic resonance (NMR) was used to determine the changes in fat mass of animals intraperitoneally treated with hIL-22 or hIL-22-ScFv (schematic shown in Fig. 3a). Fat mass, lean mass and free body fluids is shown in grams pre and post treatment. (b) One-way ANOVA, non-parametric; *P<0.01 compared to HDS-PBS control. (c) Paired t-test. ***p<0.001, compared to pre-treatment. Source data are provided as a Source Data file

a

Variant name	Description	Amino Acid Sequence<br>Underline: Signal Peptide; Grey shading: IL22; Blue shading: scFv; Red text: Fusion Linker; Blue text: Vh/Vk linker	Molecular Weight (kDa)	pI	Extinction Coefficient
**hIL-22-ScFv**	Human IL22 with (G4S)1 fusion linker and (G4S)3 Vh/Vk linker.<br><br>Expected Monomer	MGWSCIILFLVATATGVHSAPISSHCRLDKSNFQQPYITNRTFMLAKEASLADNNTDVRLIGEKLFHGVSMSERCYLMKQVLNFTLEEVLFPQSDRFQPYMQEV<br>VPFLARLSNRLSTCHIEGDDLHIQRNVQKLKDTVKKLGESGEIKAIGELDLLFMSLRNACIGGGGSEVQLLESGGGLVQPGGSLRLSCAASGFTFSSYAMSWV<br>RQAPGKGLEWVSSITAEGTHTWYADSVKGRFTISRDNSKNTLYLQMNSLRAEDTAVYYCAKTSYRFDYWGQGTLVTVSSGGGGSGGGGSGGGGSDIQMTQ<br>SPSSLSASVGDRVTITCRASQSISSYLNWYQQKPGKAPKLLIYKASR LQSGVPSRFSGSGSGTDFTLTISSLQPEDFATYYCQQKWDPPRTFGQGTKVEIKR*	42,632	8.89	**1.310**
**hIL-22-ScFv-v2**	Human IL22 with (G4S)3 fusion linker and (G4S)3 Vh/Vk linker.<br><br>Expected Monomer	MGWSCIILFLVATATGVHSAPISSHCRLDKSNFQQPYITNRTFMLAKEASLADNNTDVRLIGEKLFHGVSMSERCYLMKQVLNFTLEEVLFPQSDRFQPYMQEV<br>VPFLARLSNRLSTCHIEGDDLHIQRNVQKLKDTVKKLGESGEIKAIGELDLLFMSLRNACIGGGGSGGGGSGGGGSEVQLLESGGGLVQPGGSLRLSCAASG<br>FTFSSYAMSWVRQAPGKGLEWVSSITAEGTHTWYADSVKGRFTISRDNSKNTLYLQMNSLRAEDTAVYYCAKTSYRFDYWGQGTLVTVSSGGGGSGGGGS<br>GGGGSDIQMTQSPSSLSASVGDRVTITCRASQSISSYLNWYQQKPGKAPKLLIYKASRLQSGVPSRFSGSGSGTDFTLTISSLQPEDFATYYCQQKWDPPRT<br>FGQGTKVEIKR*	43,263	8.89	**1.291**
**hIL-22-ScFv-v3**	Human IL22 with (G4S)5 fusion linker and (G4S)3 Vh/Vk linker.<br><br>Expected Monomer	MGWSCIILFLVATATGVHSAPISSHCRLDKSNFQQPYITNRTFMLAKEASLADNNTDVRLIGEKLFHGVSMSERCYLMKQVLNFTLEEVLFPQSDRFQPYMQEV<br>VPFLARLSNRLSTCHIEGDDLHIQRNVQKLKDTVKKLGESGEIKAIGELDLLFMSLRNACIGGGGSGGGGSGGGGSGGGGSGGGGSEVQLLESGGGLVQP<br>GGSEVQLLESGGGLVQPGGSLRLSCAASGFTFSSYAMSWVRQAPGKGLEWVSSITAEGTHTWYADSVKGRFTISRDNSKNTLYLQMNSLRAEDTAVYYCAK<br>TSYRFDYWGQGTLVTVSSGGGGSGGGGSGGGGSDIQMTQSPSSLSASVGDRVTITCRASQSISSYLNWYQQKPGKAPKLLIYKASRLQSGVPSRFSGSGS<br>GTDFTLTISSLQPEDFATYYCQQKWDPPRTFGQGTKVEIKR*	**43,893**	**8.89**	**1.272**

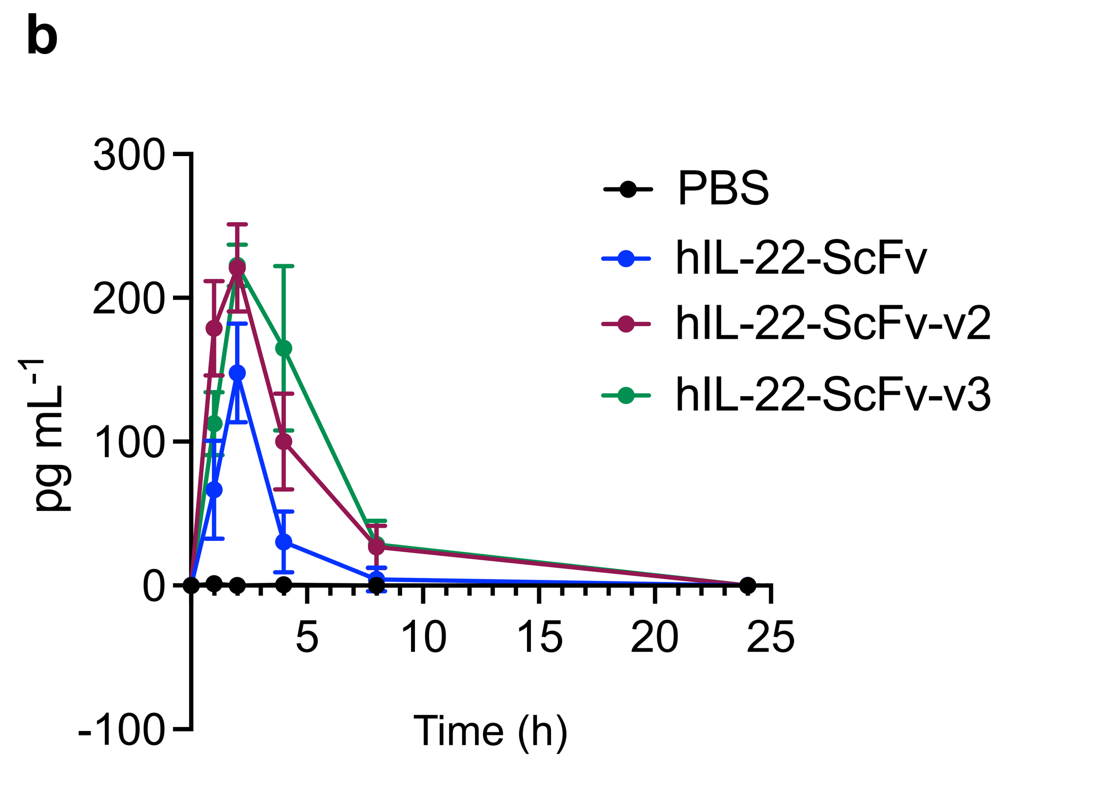

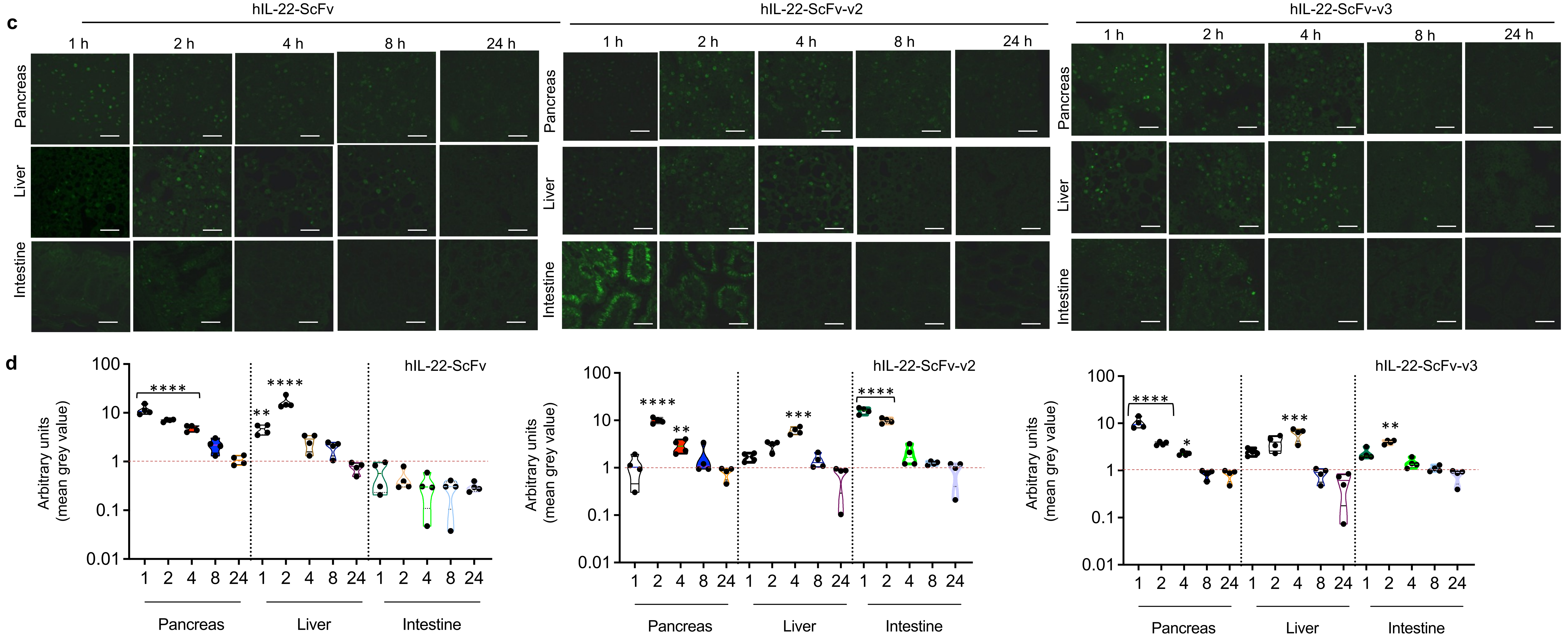

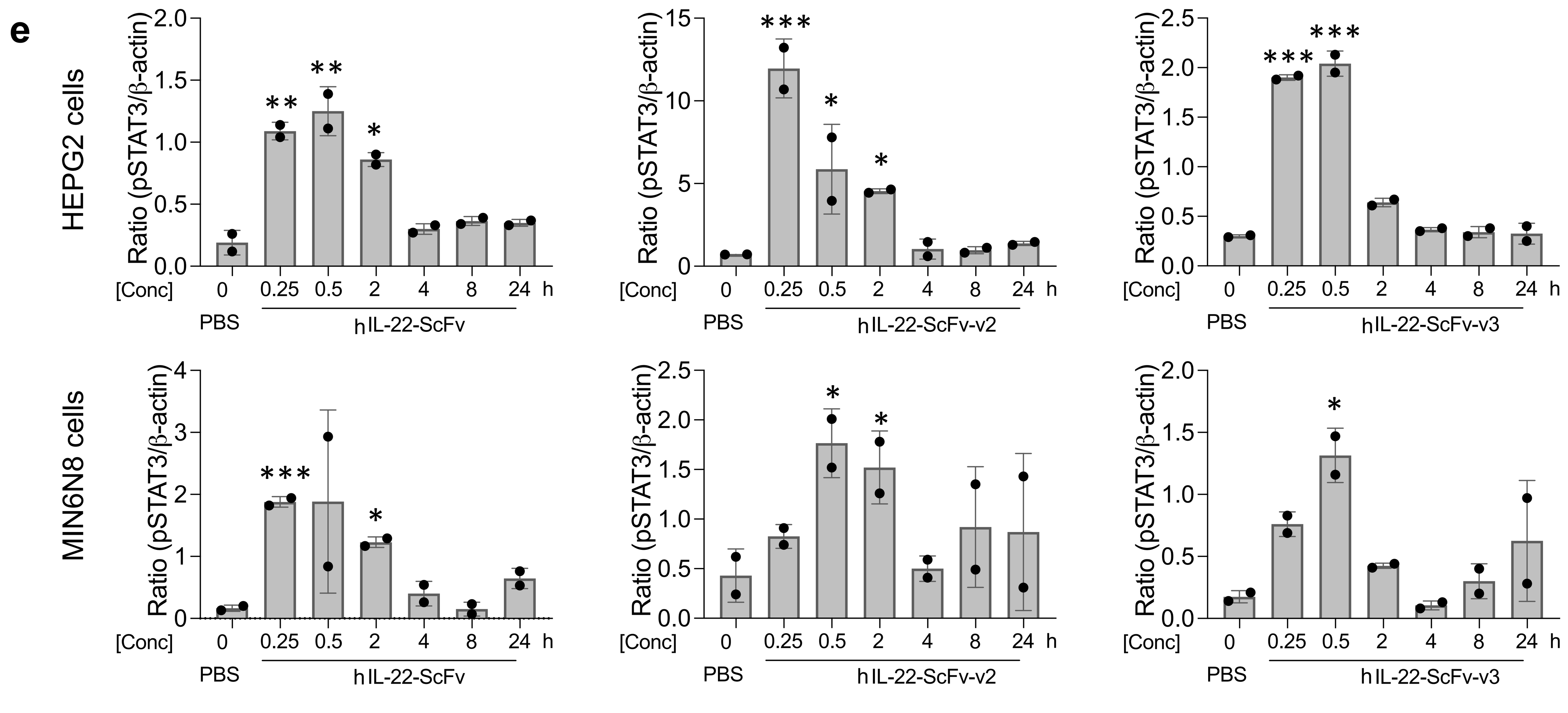

Supplementary Figure 7: **Development of IL-22-ScFv variants.** IL-22-ScFv was altered to include different linkers between IL-22 and ScFv, sequences shown in (**a**). WT C57BL/6 animals were kept on a HFD for 12 weeks and subsequently a single 6.25 pmol g^{-1} dose of hIL-22-ScFv, hIL-22-ScFv-v2 or hIL-22-ScFv-v3 subcutaneously injected. Blood was collected at 1, 2, 4, 8 and 24 h post injection, IL-22 levels were assessed using ELISA, with the IL-22-fusions were used as standards (**b**). Stat3p was assessed in the pancreas, liver and intestine at 1, 2, 4, 8 and 24 h post hIL-22-ScFv, hIL-22-ScFv-v2 and hIL-22-ScFv-v3 administration (**c**). Quantification of staining intensity is shown as mean grey value (arbitrary units) as a fold change compared to PBS treated controls (shown as a red-dashed line) (**d**). n = 4 per group, One-way ANOVA, Bonferroni's post hoc test. *p<0.05, **p<0.01, ***p<0.001, ****p<0.0001 compared to PBS. (**e**) HEPG2 (hepatoma) and MIN6N8 (insulinoma) were stimulated with IL-22-ScFv variants at 10 pmol mL^{-1} concentration and STAT3p was assessed at 6 different time points (0.25, 0.5, 1, 4, 8, 24 h) to assess the kinetics of activation compared with PBS treated cells. n = 2 per time point. PBS was used a vehicle control (con) for all experiments. ANOVA, Bonferroni's post hoc test. *p<0.05, **p<0.01, ***p<0.001, ****p<0.0001 compared to control. Scale bars: 50 μm. Source data are provided as a Source Data file

a b

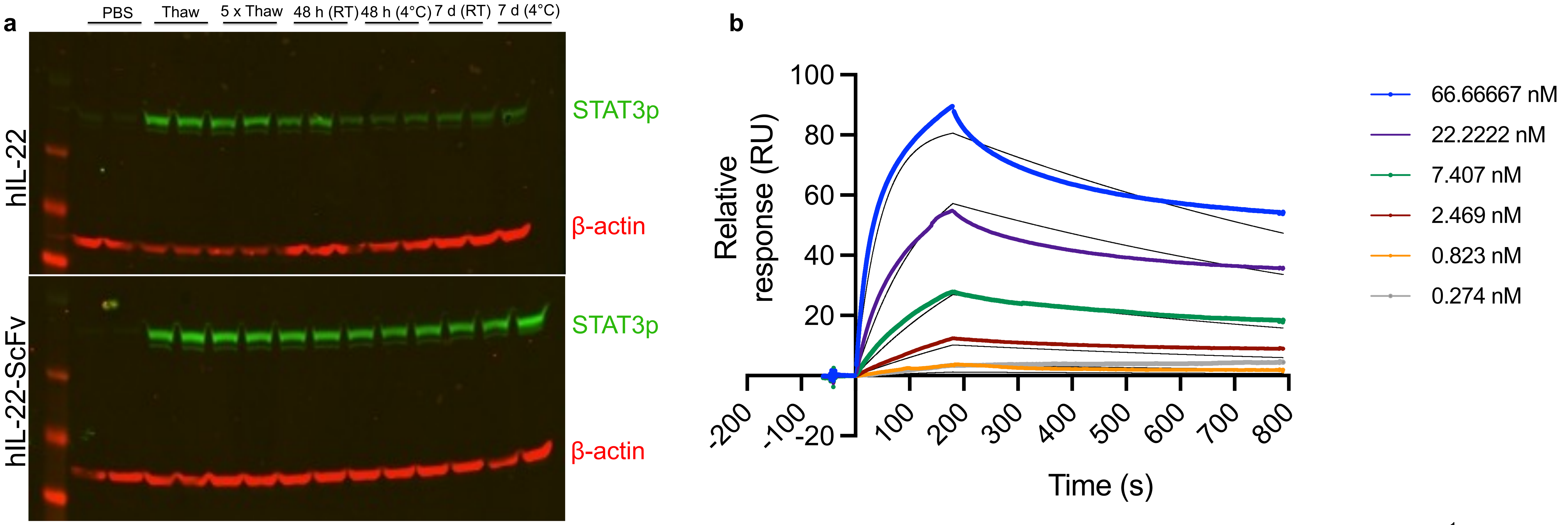

c

Summary of kinetic parameters for IL-22-ScFv variants binding to IL22Ra1				
Protein Variant	Rmax	ka ($M^{-1}sec^{-1}$)	kd (sec^{-1})	K_D (nM)
hIL-22-ScFv	88.9	2.26×10^5	7.81×10^{-4}	3.45
hIL-22-ScFv-v2	64.8	3.62×10^5	8.52×10^{-4}	2.35
hIL-22-ScFv-v3	82.9	3.58×10^5	8.14×10^{-4}	2.27
mIL-22-ScFv	85.8	3.09×10^5	8.72×10^{-4}	2.82
mIL-22-ScFv-v2	58.7	2.82×10^5	7.03×10^{-4}	2.51
mIL-22-ScFv-v3	50.6	4.09×10^5	3.94×10^{-4}	0.96

d

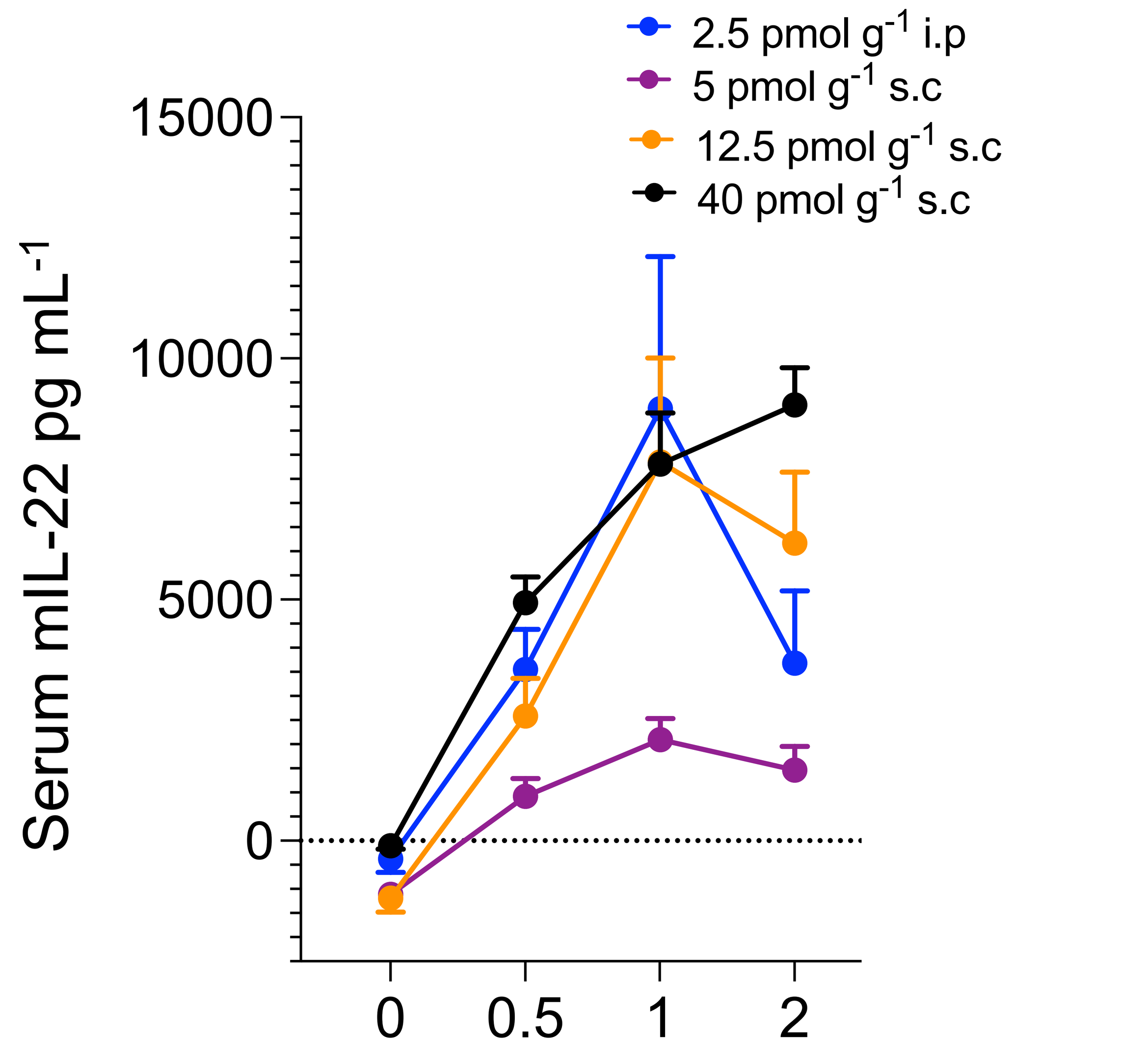

e

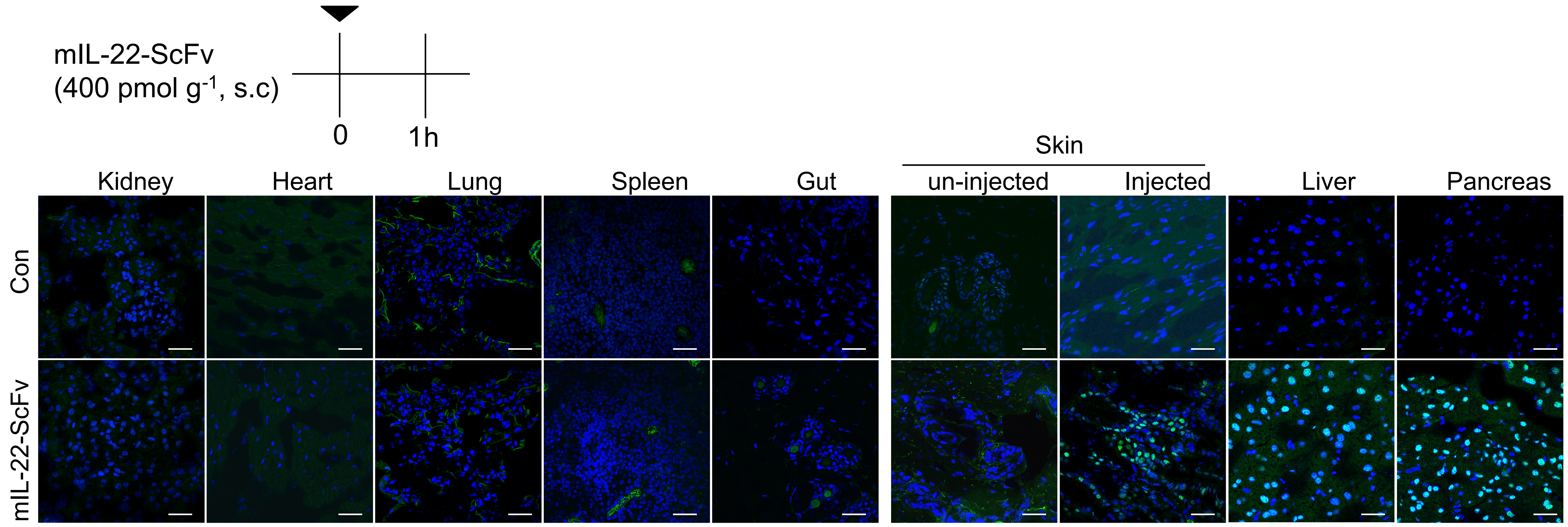

Supplementary Figure 8: IL-22-ScFv is stable, binds to IL-22RA1 and retain targeting at high concentrations. Human IL-22-ScFv and native hIL-22 was diluted at 3-5 pmol mL^{-1} concentration in PBS and incubated at various time points and temperatures as stated. HEPG2 cells were treated at the same time for 30 mins and western blots to assess STAT3p was conducted (**a**). (**b**) Binding of human IL-22-ScFv to hIL-22RA1 and (**c**) summary kinetics of all human and mouse IL-22-ScFv variants shown in Supplementary Figure 7. (**d**) WT C57BL/6 animals were injected with 5, 12.5 or 40 pmol g^{-1} of mIL-22-ScFv subcutaneously and this was compared to the therapeutic dose (2.5 pmol g^{-1}) administered i.p. (**e**) C57BL/6 animals were given 400 pmol g^{-1} of mIL-22-ScFv (s.c), tissue was isolated for Stat3p analyses by immunofluorescence compared with PBS injected controls, confirming targeting. n = 6 per group. Scale bars: 50 µm. Source data are provided as a Source Data file

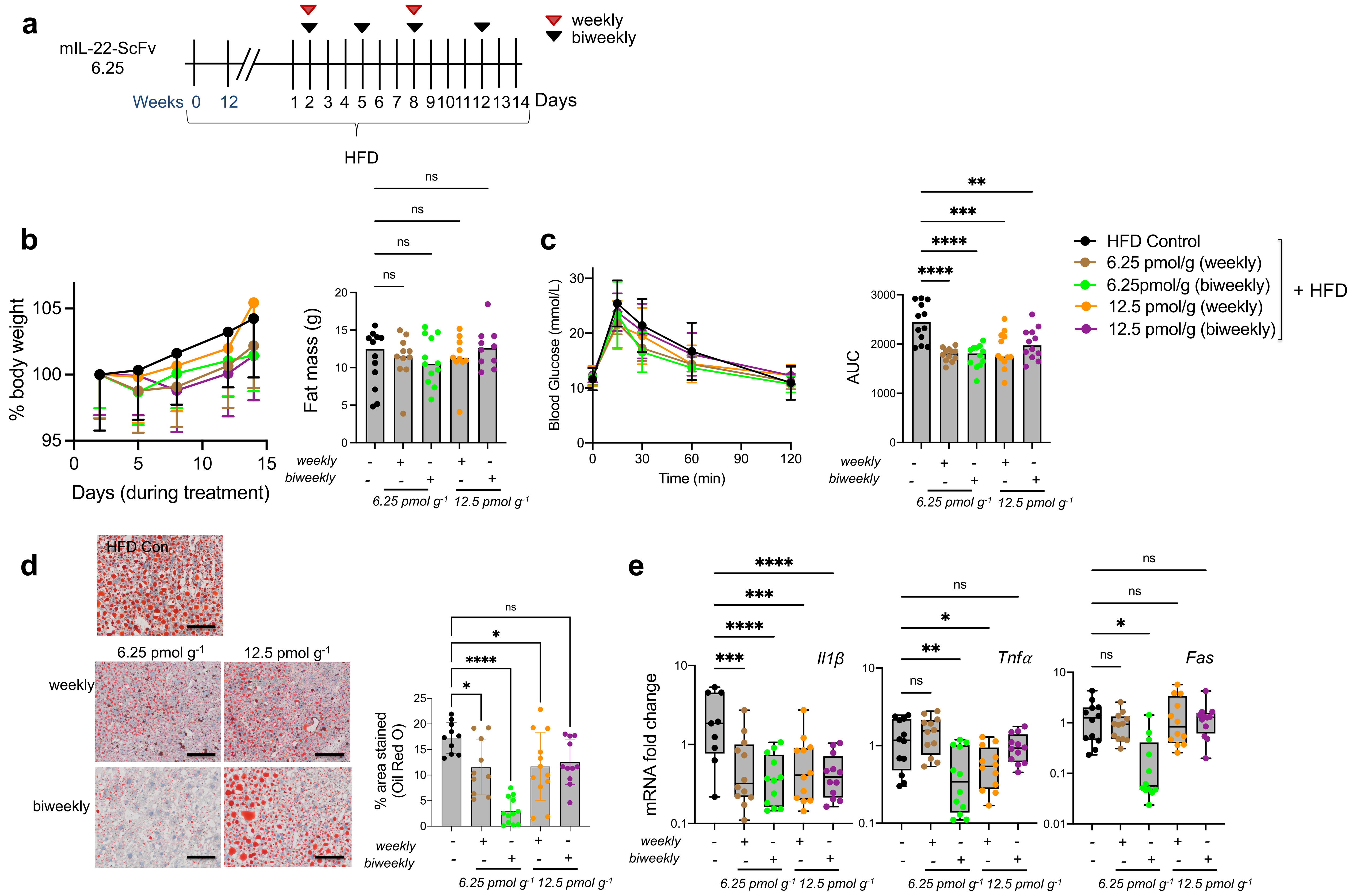

Supplementary Figure 9: mIL-22-ScFv retains targeting and efficacy when administered subcutaneously. WT C57BL/6 animals were kept on a high fat diet (HFD) for 14 weeks and then treated with mIL-22-ScFv at 6.25 or 12.5 pmol g^{-1}, s.c. weekly or biweekly (**a**, schematic) for the last two weeks. (**b**) Body weight (shown as a percentage) and fat mass (measured via NMR) during the treatment, (**c**) Oral glucose tolerance test was assessed, AUC shown after 14 days of treatment. (**d**) Oil Red O staining of liver and percentage of area stained compared with HFD controls; scale bar: 100 µm. (**e**) Hepatic gene expression analyses of *Il1b, Tnfa* and *Fas* in animals after 14 days of treatment. n = 12 per group. One-way ANOVA, Bonferroni's post hoc test. *p<0.05, **p<0.01, ***p<0.001, ****p<0.0001 compared to HFD control. Source data are provided as a Source Data file.

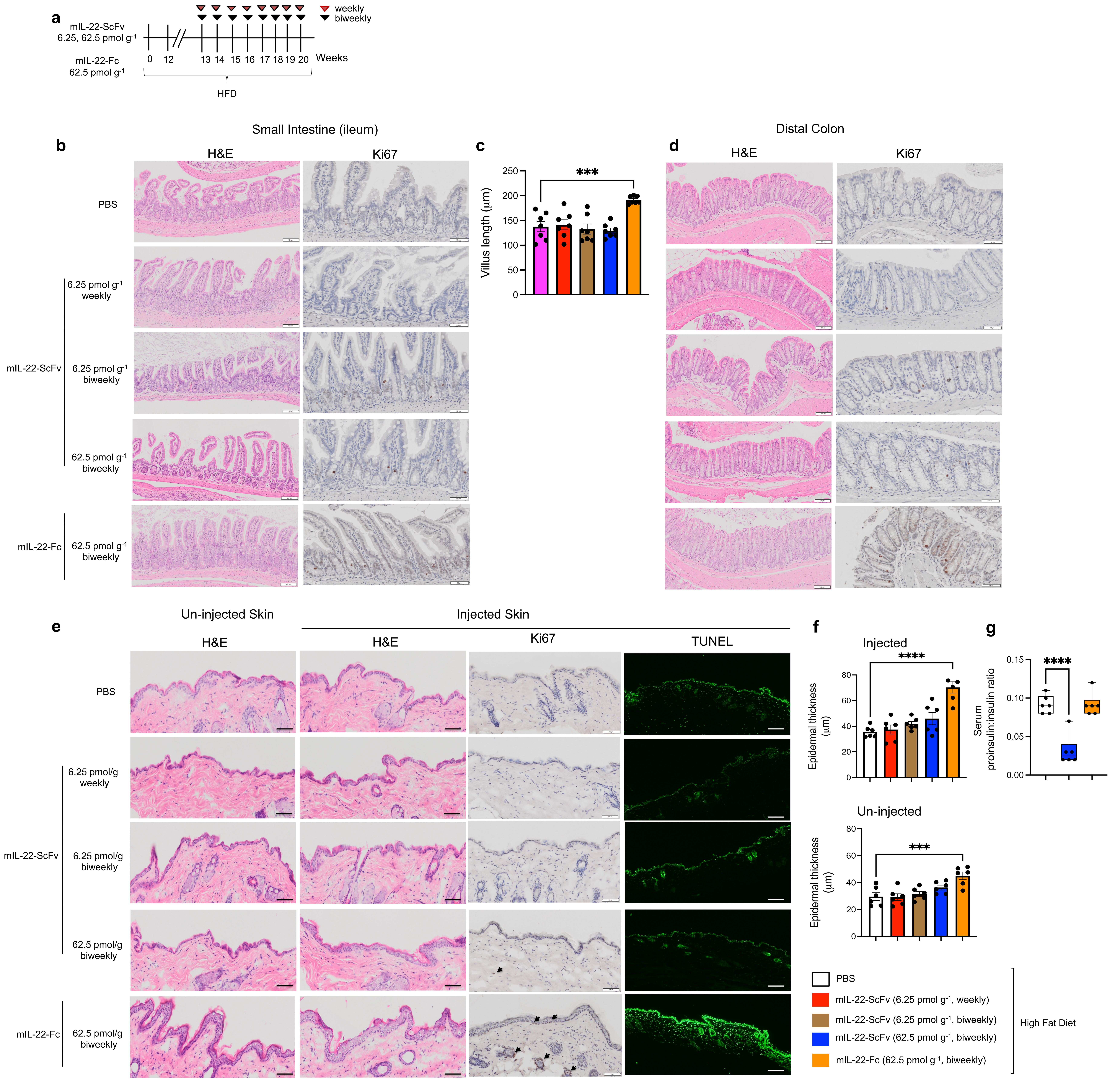

Supplementary Figure 10: WT C57BL/6 animals were kept on a high fat diet (HFD) for 20 weeks and then treated for the last 8 weeks of diet with mIL-22-ScFv at 6.25 or 62.5 pmol g^{-1}, s.c. weekly or biweekly or mIL-22-Fc at 62.5 pmol g^{-1}, biweekly, s.c (**a**, schematic). (**b**) H&E (scale bars: 100 µm) and Ki67 staining (scale bar: 50 µm) and, (**c**) Villus length measurements in the small intestine. (**d**) H&E (scale bar: 100 µm) and Ki67 staining (scale bar: 50 µm) in the distal colon. (**e**) H&E staining (scale bar: 50 µm) of uninjected and injected skin, Ki67 staining (scale bar: 50 µm) showing proliferation and TUNEL staining (scale bar: 100 µm) showing apoptosis. (**f**) Quantification of epidermis thickness in all treated HFD animals compared to HFD animals treated with PBS. (**g**) Proinsulin:insulin ratio of HFD animals treated with PBS, or mIL-22-ScFv or mIL-22-Fc at 62.5 pmol g^{-1}, biweekly, s.c. n = 6 per group. One-way ANOVA, Bonferroni's post hoc test. *p<0.05, **p<0.01, ***p<0.001, ****p<0.0001 compared to HFD control. Source data are provided as a Source Data file.

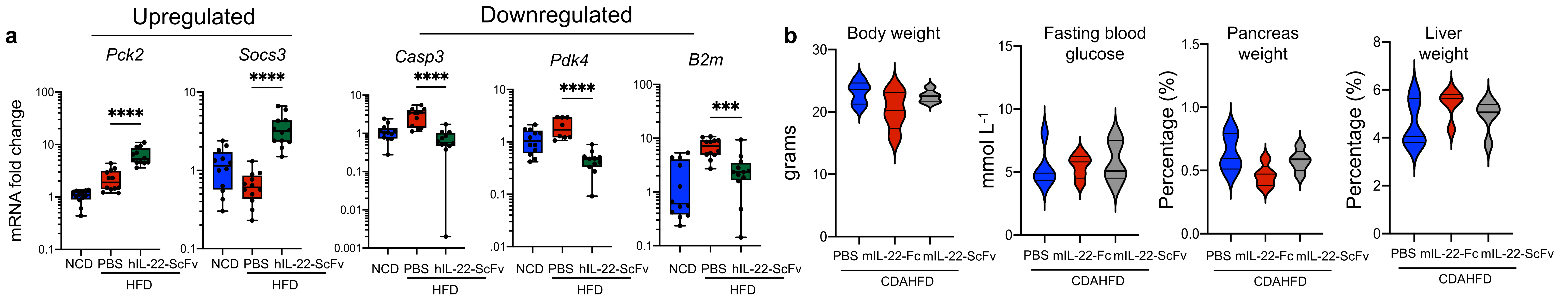

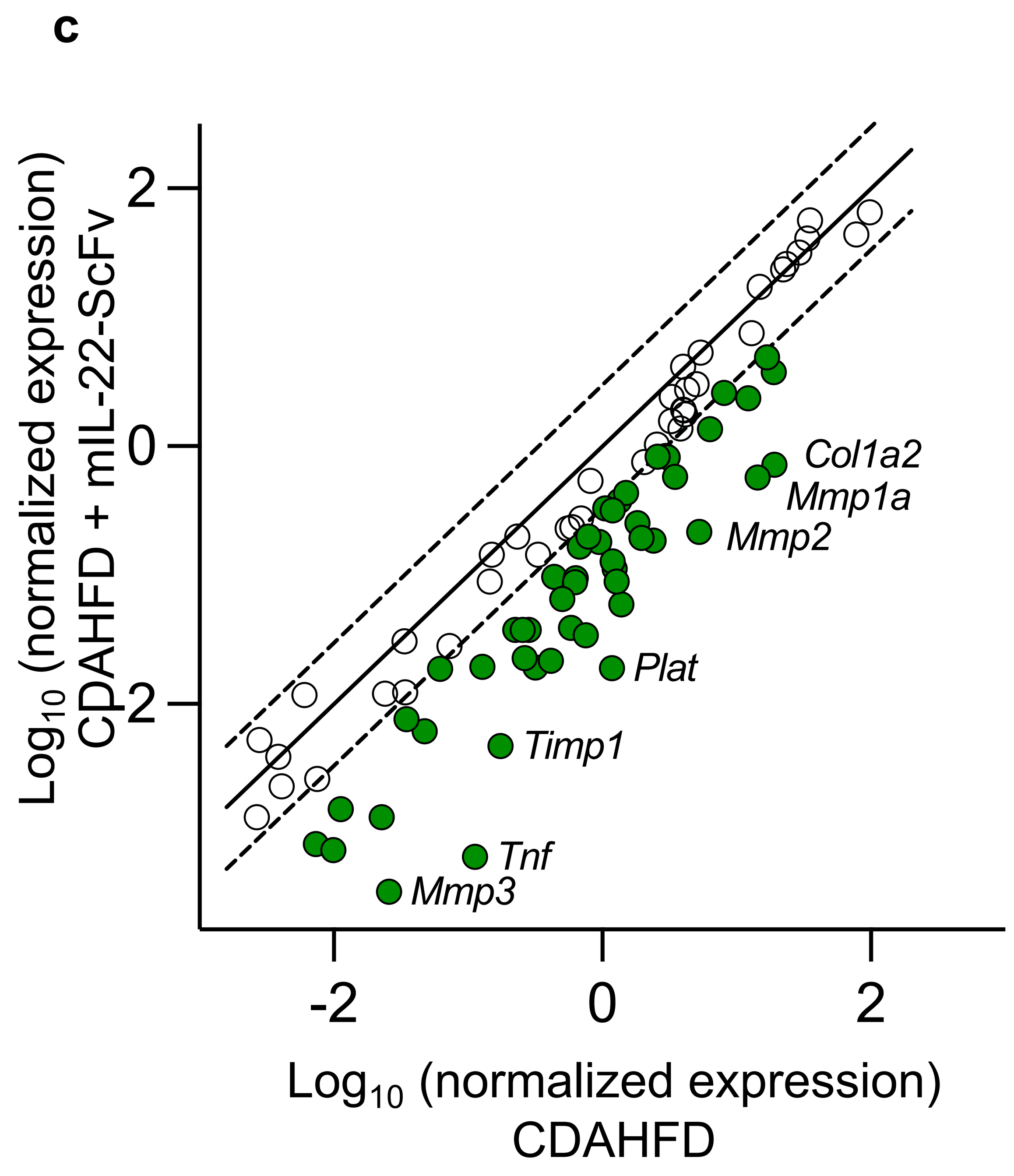

d

	Pro-fibrotic		Inflammatory		Signal Transduction		Cell adhesion	
1	Mmp3	-73.2	Il5	-17.12	Thbs2	-23.5	Itga2	-16.7
2	Timp1	-36.8	Ccl3	-14.99	Thbs1	-9.42	Itgb8	-11.1
3	Col1a2	-26.6	Il10	-13.47	Cav 1	-5.26	Itgb6	-6.67
4	Mmp1a	-25.3	Ifng	-8.9	Dcn	-5.06	Itga3	-3.95
5	Mmp2	-24.6	Tgfb3	-7.74	Jun	-3.54	Itgb5	-3.45
6	Lox	-21.8	Il13	-7.39			Itgav	-3.11
7	Timp2	-14.5	Tgfb2	-6.77				
8	Col3a1	-12.9	Ccr2	-6.6				
9	Mmp13	-11.7	Cxcr4	-5.97				
10	Pdgfb	-10	Tgfb1	-4.63				
11	Mmp8	-7.73	Tgfbr2	-3.77				
12	Mmp9	-7.58	Tgif1	-3.34				
13	Ltbp1	-7.12						
14	Serpinh1	-6.08						
15	Timp3	-5.21						
16	Serpine1	-4.57						
17	Mmp14	-3.75						
18	Acta2 (α-SMA)	-3.53						
19	Pdgfa	-3.2						

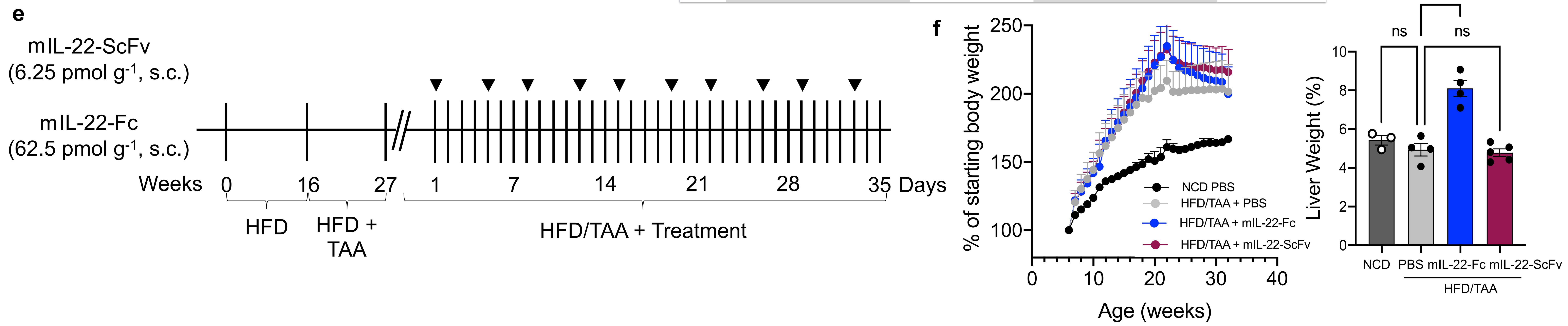

g

HFD/TAA model (Control vs mIL-22-ScFv Treated)	
Bmp7	-2.12
Edn1	-2.10
Grem1	-10.54
Il13	-3.05
Mmp3	-15.20
Tgfb2	-2.16
Timp4	-2.39

Supplementary Figure 11: IL-22-ScFv downregulates pro-fibrotic and inflammatory genes. (**a**) Gene expression analyses of HFD (12 weeks) and HFD animals treated with hIL-22-ScFv (biweekly, 2 weeks); validation of genes identified in Fatty Liver RT^2 Profiler PCR Array (**Fig 4a**). (**b**) Body weight, fasting blood glucose levels and pancreas and liver weight from C57BL/6 animals placed on a choline-deficient, L-amino acid-defined, high-fat diet (CDAHFD) for 11 weeks and treated biweekly with mIL-22-Fc (62.5 pmol g^{-1}) or mIL-22-ScFv (6.25 pmol g^{-1}) s.c. for the last 5 weeks of diet. (**c**) Fibrosis RT^2 Profiler PCR Array comparing differences between CDAHFD (PBS treated) and mIL-22-ScFv treated animals. (**d**) Genes identified to be downregulated by IL-22-ScFv treatment in the CDAHFD model. (**e**) Schematic showing the regime of the experiment in C57BL/6 obese animals (16 weeks of HFD), subsequently challenged with HFD and TAA for another 16 weeks, with treatments in the last 5 weeks, with s.c. mIL-22-ScFv (6.25 pmol g^{-1}) or mIL-22-Fc (62.5 pmol g^{-1}). (**f**) Changes in body weight and liver weight in the HFD/TAA model. (**g**) Genes identified to be downregulated using fibrosis RT^2 Profiler PCR Array comparing differences between HFD/TAA (PBS treated) and mIL-22-ScFv treated animals. n = 4-12. One-way ANOVA, Bonferroni's post hoc test. *$p<0.05$, **$p<0.01$, ***$p<0.001$, ****$p<0.0001$. Source data are provided as a Source Data file.

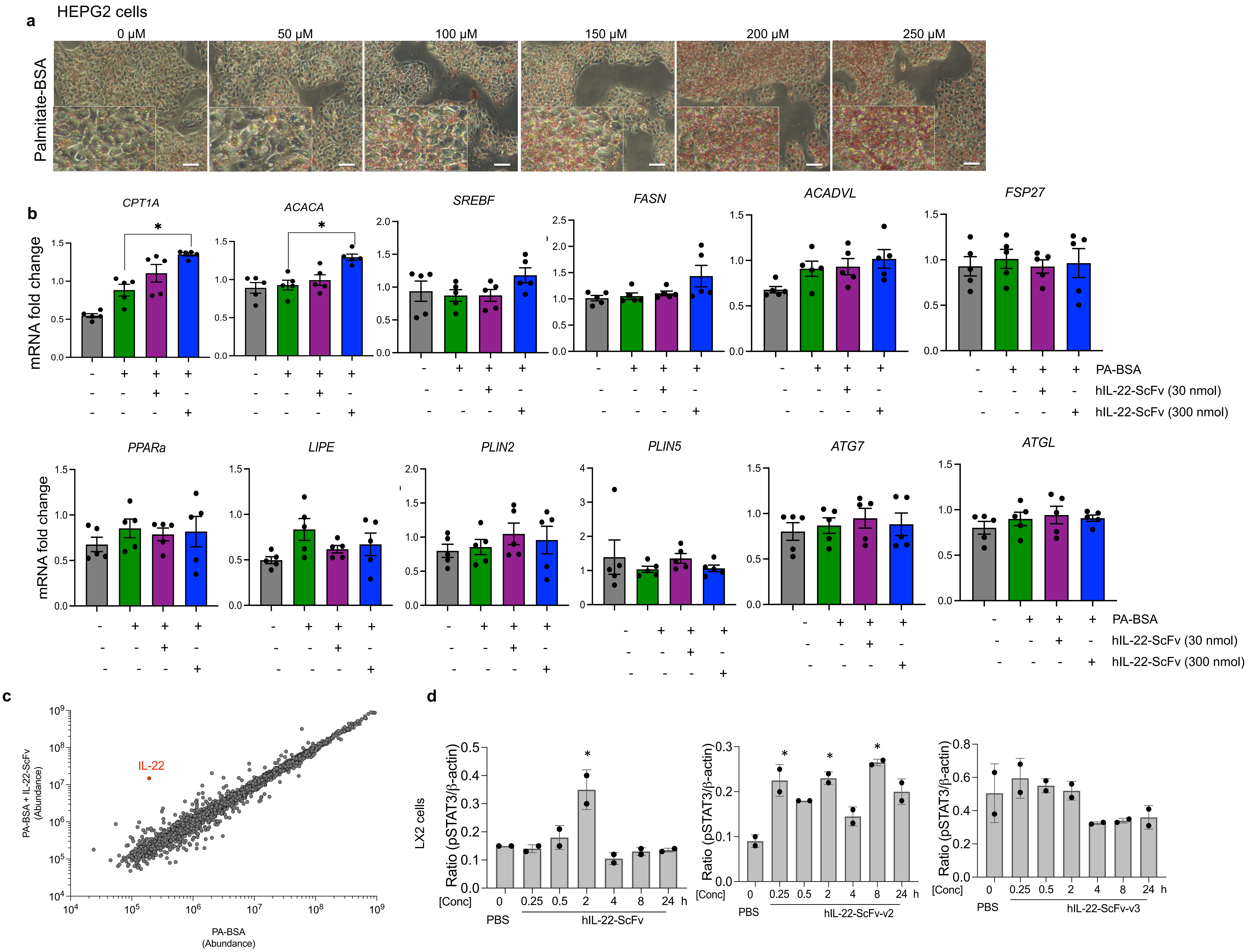

Supplementary Figure 12: hIL-22-ScFv effect on HEPG2, and LX2 cells. (**a**) HEPG2 cells were treated with increasing concentrations of palmitate and oil red o staining used to determine changes in lipid accumulation; inset x 10 magnification; scale bar: 200 µm. (**b**) RT-PCR used to assess the expression of lipolysis genes in HEPG2 cells were treated with Palmitate (200 µM) to induce lipid accumulation and treated with hIL-22-ScFv (30 or 300 nmol mL^{-1}); n = 4. c, Palmitate stimulated HEPG2 cells were treated with IL-22-ScFv or vehicle only and analysed using mass spectroscopy; which led to identifying 5480 proteins with high confidence **d**, LX2 cells treated with 30 nmol mL^{-1} of hIL-22-ScFv (and variants, described in Supplementary Figure 8) and STAT3p assessed at different times as outlined on the x-axis. One-way ANOVA, Bonferroni's post hoc test. *p<0.05. Source data are provided as a Source Data file

Supplementary Table 1: Demographic information about human pancreatic islet donors.

Donor	Sex	Age (Range)	BMI	History
1	F	60 - 65	35	No substantive history
2	M	60 - 65	29	No substantive history
3	M	55 - 60	26	On medication for Hypertension, Smoker
4	F	60 - 65	27	No substantive history

Supplementary Table 2: Human and mouse primer sequences.

Human Primers		
**Gene Name**	**Forward Primer**	**Reverse Primer**
ACACA	TCACACCTGAAGACCTTAAAGCC	AGCCCACACTGCTTGTACTG
ACADVL	ACGATCCCGCCAAGAATGAC	TTGAAACCGATGCTCTGATGG
ATG7	CTGCCAGCTCGCTTAACATTG	CTTGTTGAGGAGTACAGGGTTTT
ATGL	ATGGTGGCATTTCAGACAACC	CGGACAGATGTCACTCTCGC
B-ACTIN	CCTGTACGCCAACACAGTGC	ATACTCCTGCTTGCTGATCC
CPT1A	ATGCGCTACTCCCTGAAAGTG	CGGTGGTTTCTTGGTCGGT
FASN	AAGGACCTGTCTAGGTTTGATGC	TGGCTTCATAGGTGACTTCCA
FSP27	ATTGATGTGGCCCGTGTAACG	CAGCAGTGCAGATCATAGGAAA
LIPE	CCTCAGCCTTCTTCCATGAG	CACAGCTGCATTGCCATAGT
PLIN2	TTGCAGTTGCCAATACCTATGC	CCAGTCACAGTAGTCGTCACA
PLIN5	AAGGCCCTGAAGTGGGTTC	GCATGTGGTCTATCAGCTCCA
PPARa	TTCGCAATCCATCGGCGAG	CCACAGGATAAGTCACCGAGG
SREBF	GCCCCTGTAACGACCACTG	CAGCGAGTCTGCCTTGATG
sXBP-1	GAGTCCGCAGCAGGTGC	CAAAAGGATATCAGACTCAGAATCTGAA

Mouse Primers		
**Gene Name**	**Forward Primer**	**Reverse Primer**
B2m	CCCCACTGAGACTGATACATACG	CGATCCCAGTAGACGGTCTTG
Cd68	TGTCTGATCTTGCTAGGACCG	GAGAGTAACGGCCTTTTTGTGA
Col1a1	GCTCCTCTTAGGGGCCACT	CCACGTCTCACCATTGGGG
Col1a2	GTAACTTCGTGCCTAGCAACA	CCTTTGTCAGAATACTGAGCAGC
Col3a1	CTGTAACATGGAAACTGGGGAAA	CCATAGCTGAACTGAAAACCACC
Cpt1a	CTCCGCCTGAGCCATGAAG	CACCAGTGATGATGCCATTCT
Elovl6	GAAAAGCAGTTCAACGAGAACG	AGATGCCGACCACCAAAGATA
Fas	GGCATCATTGGGCACTCCTT	GCTGCAAGCACAGCCTCTCT
Grp78	CGCCTCATCGGACGCACTTGG	GCTTGCCGCTGTGCATCATTGA
Hmgcr	AGCTTGCCCGAATTGTATGTG	TCTGTTGTGAACCATGTGACTTC
Il1b	AGAGTTACACTGCCTTTGCCATCC	CCACGTCAATCTTTCCTCTTGCTT
Ldlr	AGTGGCCCCGAATCATTGAC	CTAACTAAACACCAGACAGAGGC
Mmp2	CAAGTTCCCCGGCGATGTC	TTCTGGTCAAGGTCACCTGTC
Nos2	CAGCTGGGCTGTACAAACCTT	CATTGGAAGTGAAGCGTTTCG
Ppia	CAAATGCTGGACCAAACACAAACG	GTTCATGCCTTCTTTCACCTTCCC
Srebp1c	GGAGCCATGGATTGCACATT	GGCCCGGGAAGTCACTGT
sXbp1	GAGTCCGCAGCAGGTGC	CAAAAGGATATCAGACTCAGAATCTGAA
Tata	CCCTCTTCAAAAACTTCTCCAC	ACCAACAATCACCAACAGCA
Timp2	TCAGAGCCAAAGCAGTGAGC	GCCGTGTAGATAAACTCGATGTC
Tnfa	CATCTTCTCAAAATTCGAGTGACAA	TGGGAGTAGACAAGGTACAACCC

Uncropped Western Blot Images

Reducing (+ BME) and non-reducing (- BME) SDS-PAGE of purified IL-22-fusions

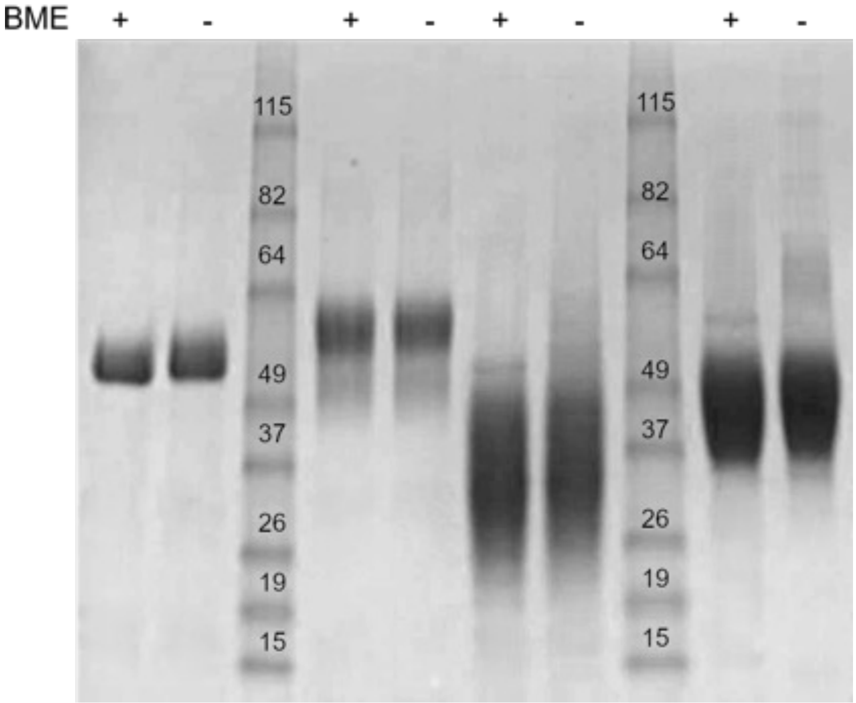

Uncropped western blot – Supplementary Fig. 4b

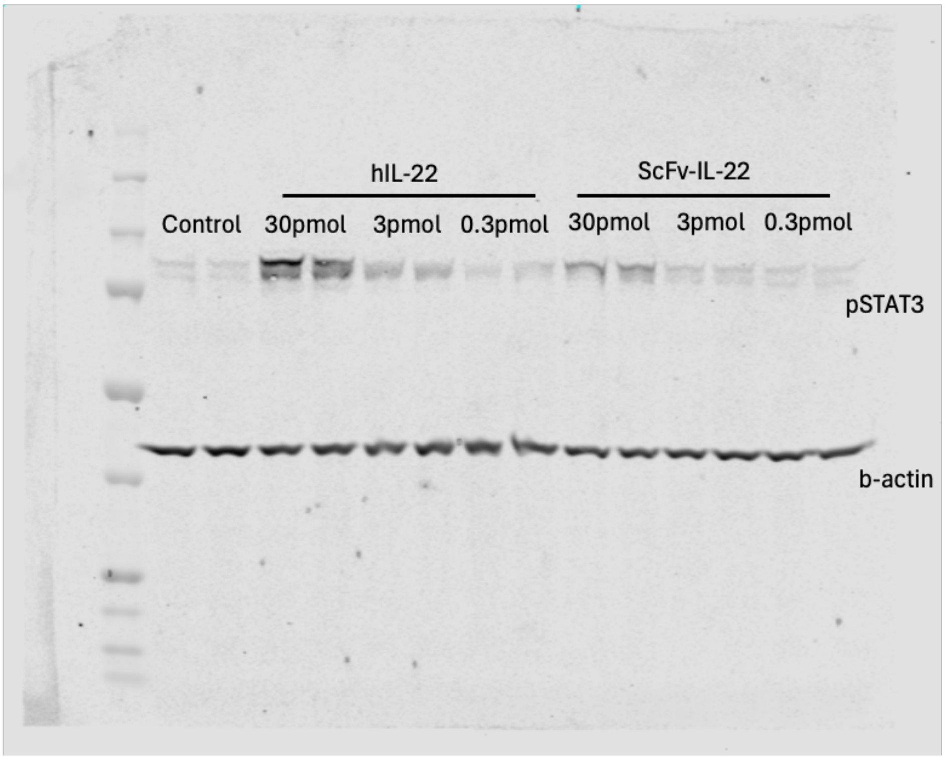

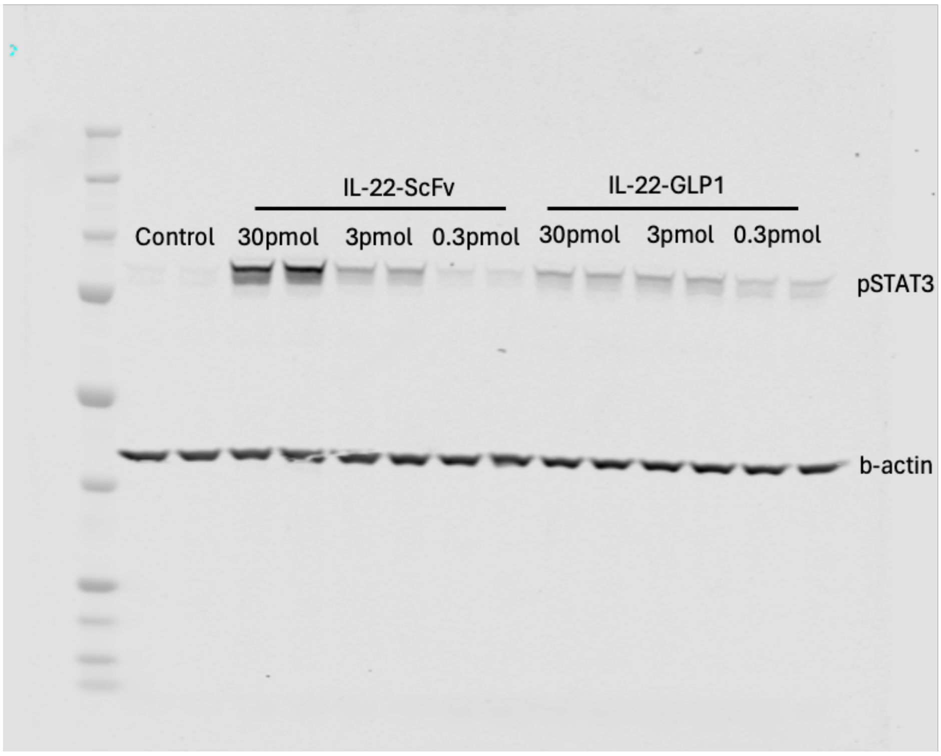

Uncropped western blots – Supplementary Fig. 5a

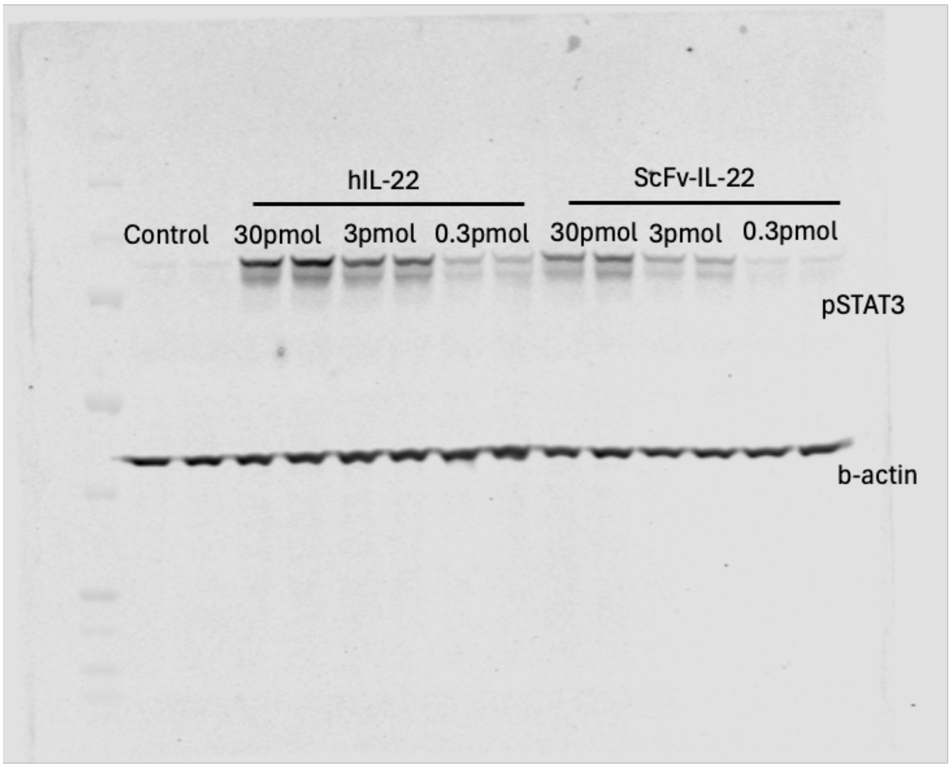

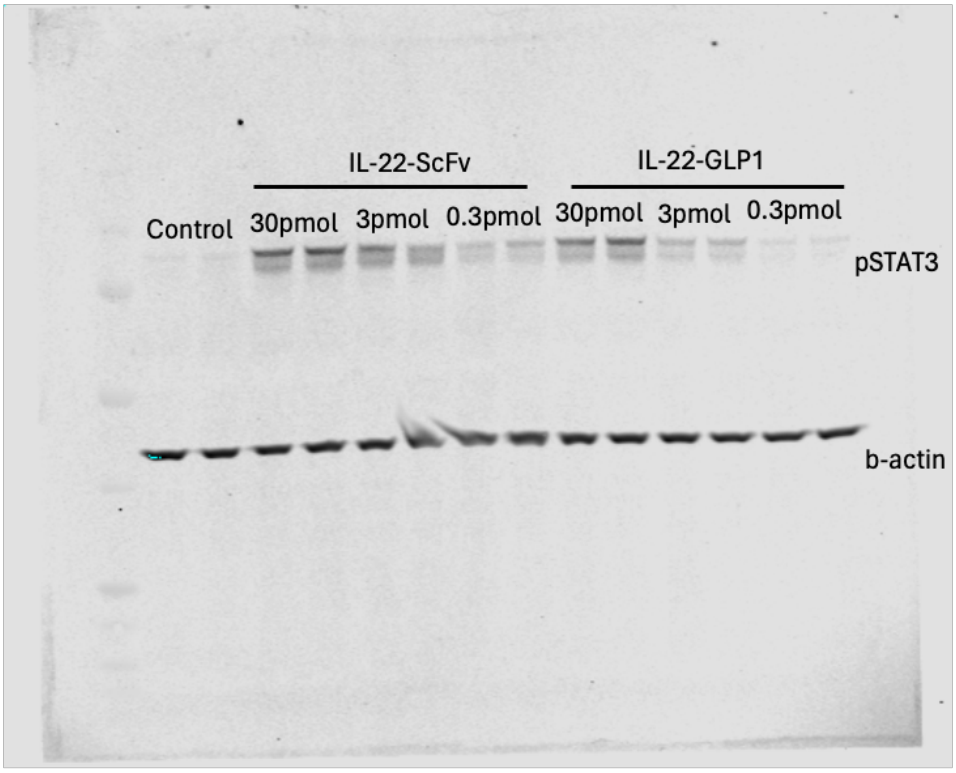

Uncropped western blots – Supplementary Fig. 5b

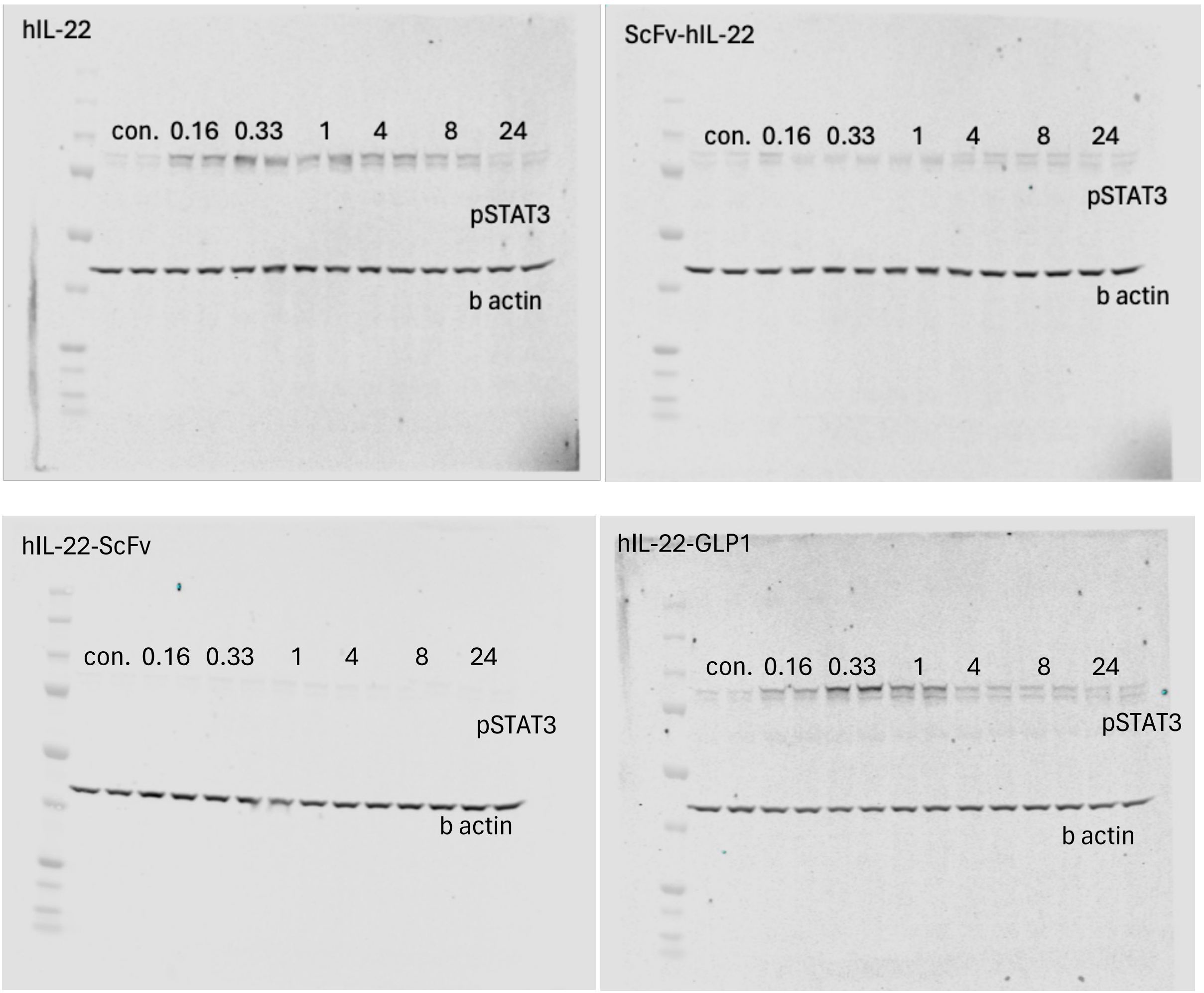

Uncropped western blots – Supplementary Fig. 5c-f

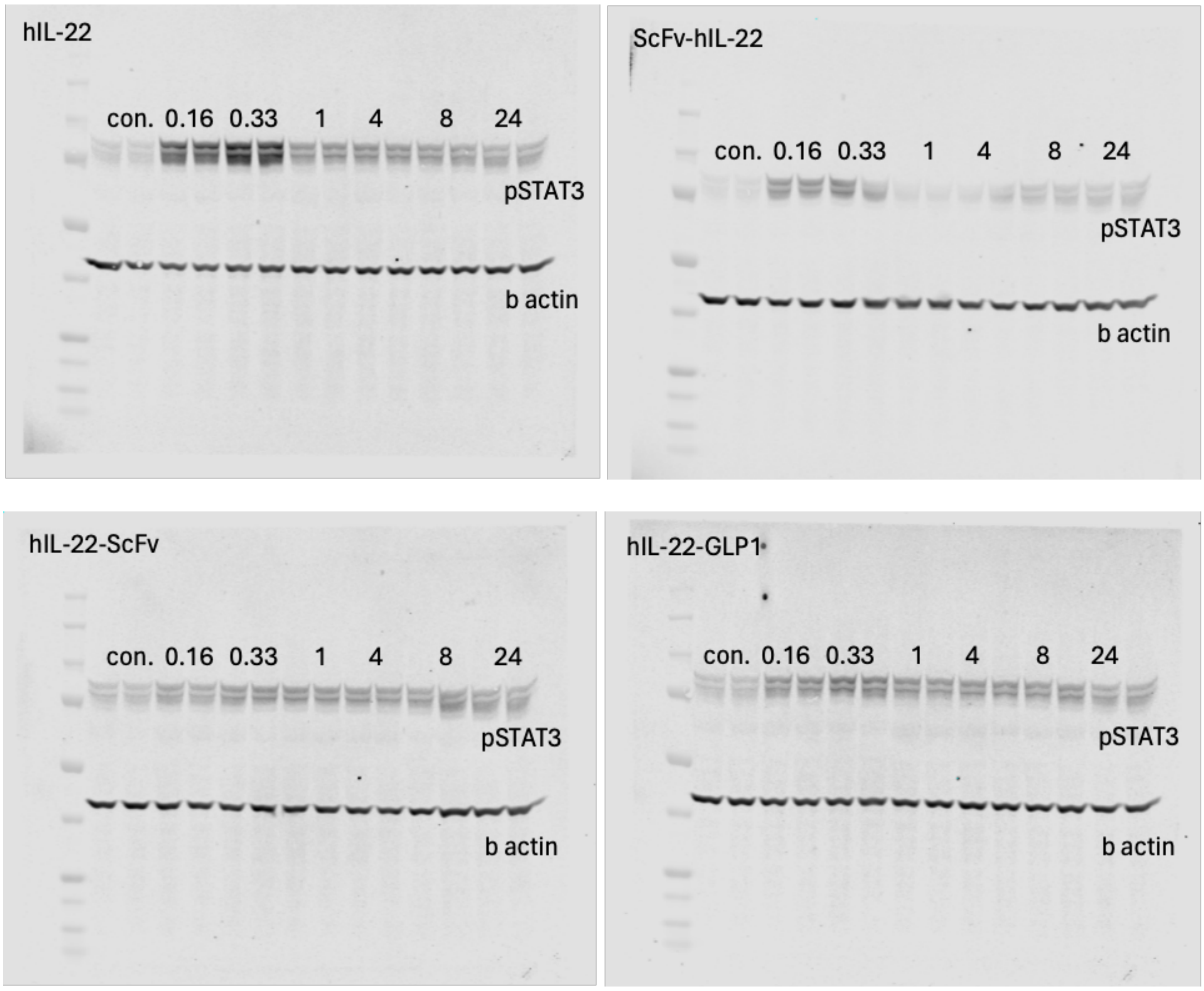

Uncropped western blots – Supplementary Fig. 5g-j

1. IL-22-ScFv

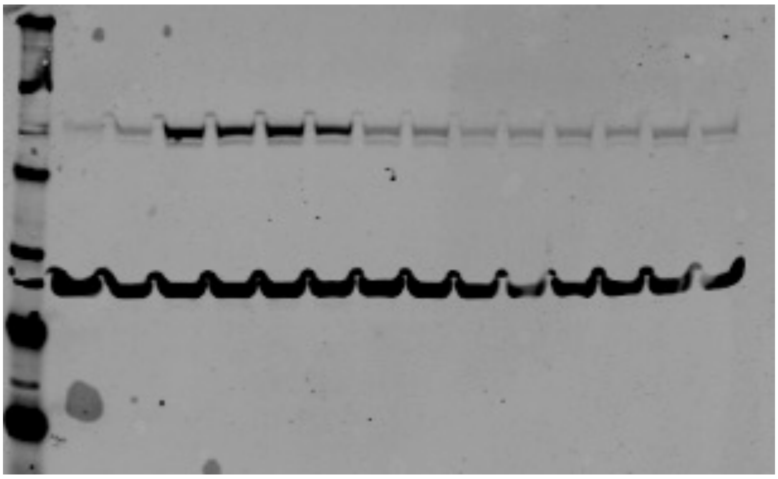

Top-pSTAT3

Bottom-β-actin

Lane 1-2: PBS

Lane 3-4: IL-22-ScFv 15mins

Lane 5-6: IL-22-ScFv -30mins

Lane 7-8: IL-22-ScFv 2hrs

Lane 9-10: IL-22-ScFv -4hrs

Lane 11-12: IL-22-ScFv -8hrs

Lane 13-14: IL-22-ScFv -24hrs

2. IL-22-ScFv-v2

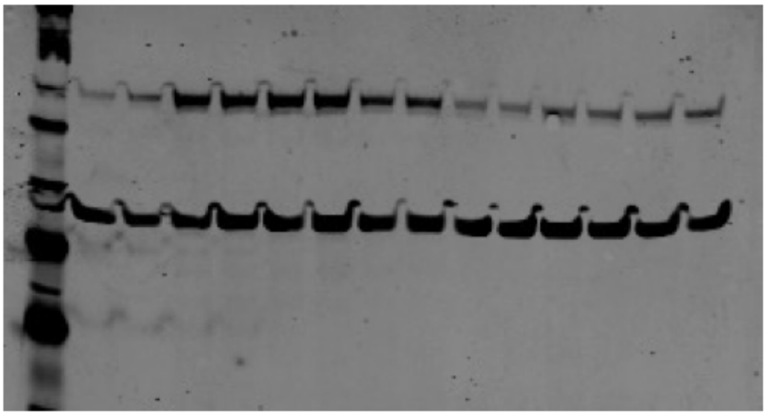

- Top-pSTAT3
- Bottom-β-actin
- Lane 1-2: PBS
- Lane 3-4: IL-22-ScFv-v2 15mins
- Lane 5-6: IL-22-ScFv-v2 -30mins
- Lane 7-8: IL-22-ScFv-v2 2hrs
- Lane 9-10: IL-22-ScFv-v2 -4hrs
- Lane 11-12: IL-22-ScFv-v2 -8hrs
- Lane 13-14: IL-22-ScFv-v2 -24hrs

3. IL-22-ScFv-v3

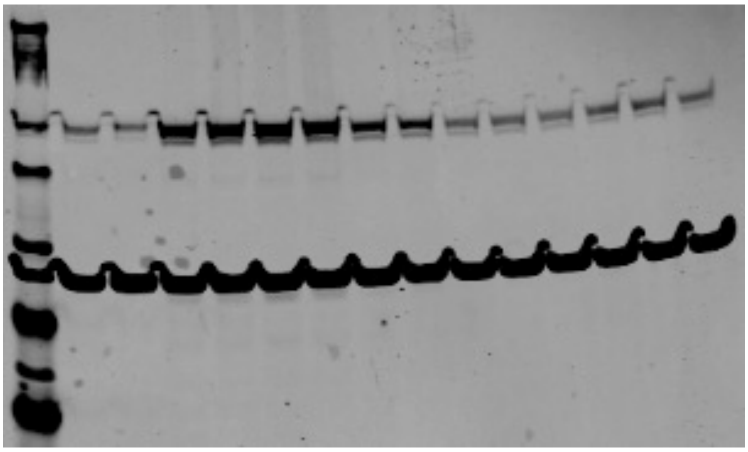

- Top-pSTAT3
- Bottom-β-actin
- Lane 1-2: PBS
- Lane 3-4: IL-22-ScFv-v3 15mins
- Lane 5-6: IL-22-ScFv-v3 -30mins
- Lane 7-8: IL-22-ScFv-v3 2hrs
- Lane 9-10: IL-22-ScFv-v3 -4hrs
- Lane 11-12: IL-22-ScFv-v3 -8hrs
- Lane 13-14: IL-22-ScFv-v3 -24hrs

1. IL-22-ScFv

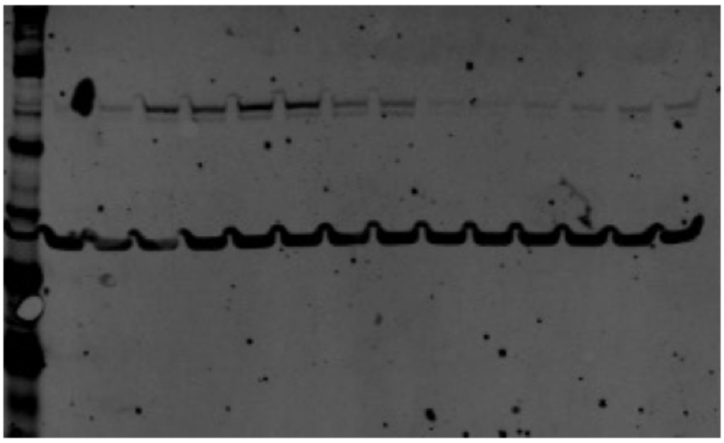

- Top-pSTAT3
- Bottom-β-actin
- Lane 1-2: PBS
- Lane 3-4: IL-22-ScFv-15mins
- Lane 5-6: IL-22-ScFv--30mins
- Lane 7-8: IL-22-ScFv--2hrs
- Lane 9-10: IL-22-ScFv-4hrs
- Lane 11-12: IL-22-ScFv--8hrs
- Lane 13-14: IL-22-ScFv--24hrs

2. IL-22-ScFv-v2

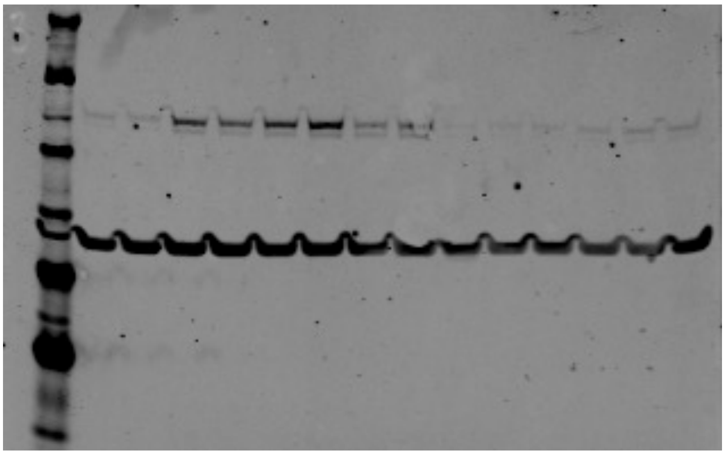

- Top-pSTAT3
- Bottom-β-actin
- Lane 1-2: PBS
- Lane 3-4: IL-22-ScFv-v2-15mins
- Lane 5-6: IL-22-ScFv-v2-30mins
- Lane 7-8: IL-22-ScFv-v2 -2hrs
- Lane 9-10: IL-22-ScFv-v2 -4hrs
- Lane 11-12: IL-22-ScFv-v2-8hrs
- Lane 13-14: IL-22-ScFv-v2-24hrs

3. IL-22-ScFv-v3

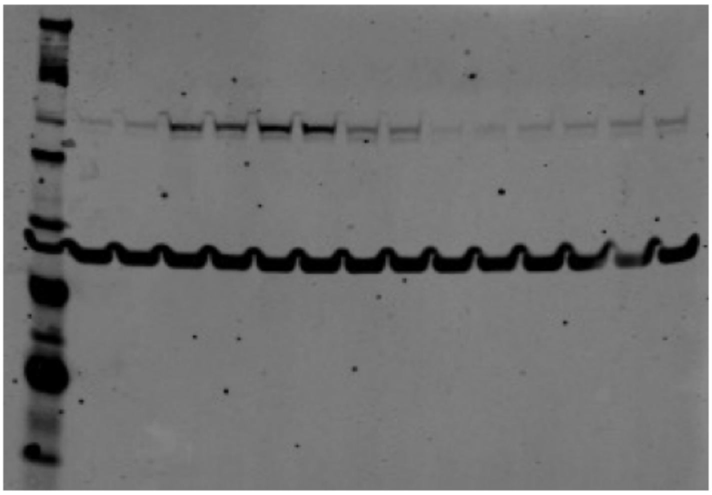

- Top-pSTAT3
- Bottom-β-actin
- Lane 1-2: PBS
- Lane 3-4: IL-22-ScFv-v3-15mins
- Lane 5-6: IL-22-ScFv-v3-30mins
- Lane 7-8: IL-22-ScFv-v3 -2hrs
- Lane 9-10: IL-22-ScFv-v3 -4hrs
- Lane 11-12: IL-22-ScFv-v3-8hrs
- Lane 13-14: IL-22-ScFv-v3-24hrs

Uncropped western blots – Supplementary Fig. 7e

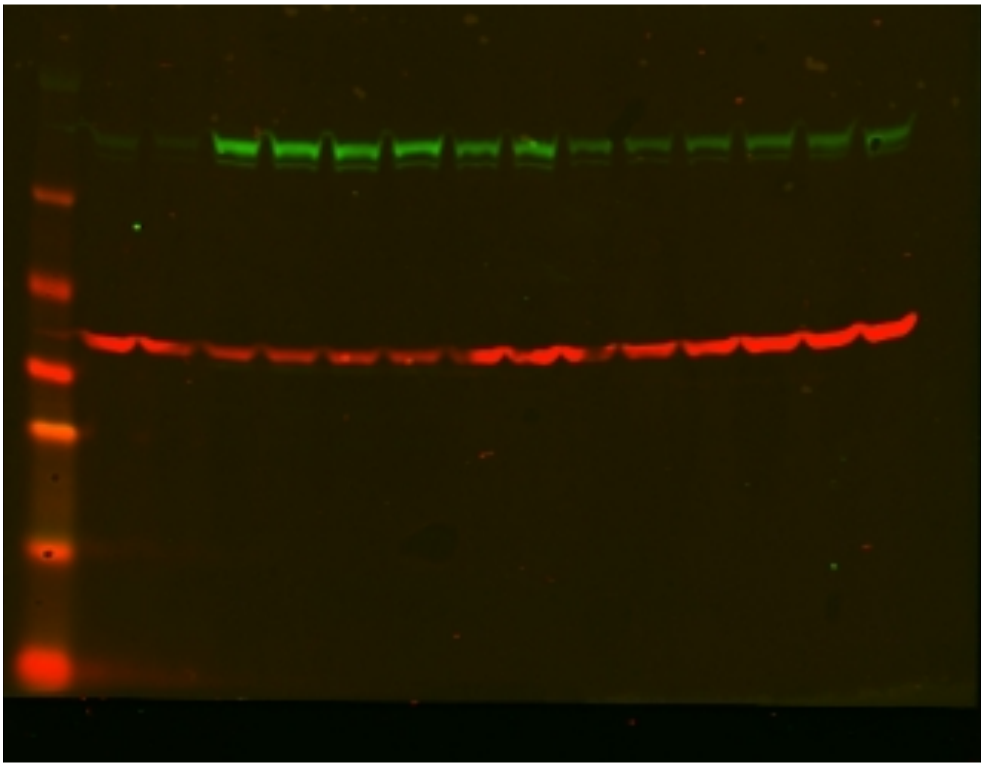

Green-pSTAT3

Red-β-actin

Lane 1-2: PBS

Lane 3-4: Fresh Thaw

Lane 5-6: Freeze Thaw 5X

Lane 7-8: 48hrs RT

Lane 9-10: 48hrs 4°C

Lane 11-12: 7 days RT

Lane 13-14: 7 days 4°C

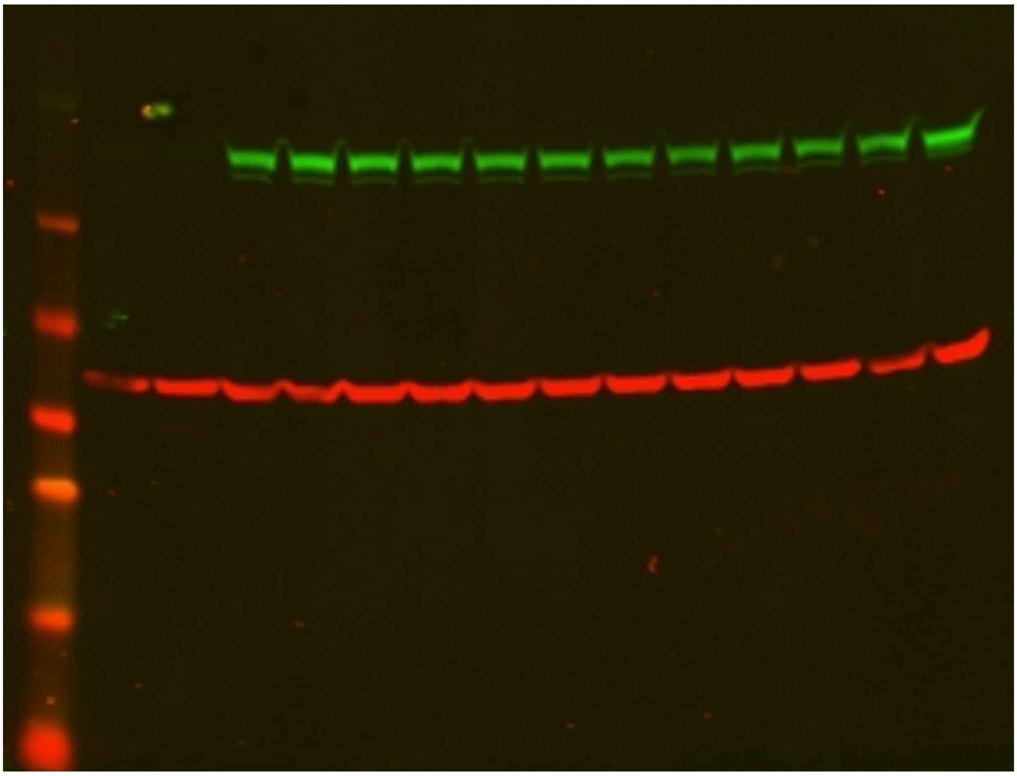

Green-pSTAT3

Red-β-actin

Lane 1-2: PBS

Lane 3-4: Fresh

Lane 5-6: Freez

Lane 7-8: 48hrs

Uncropped western blots – Supplementary Fig. 8a

1. IL-22-ScFv

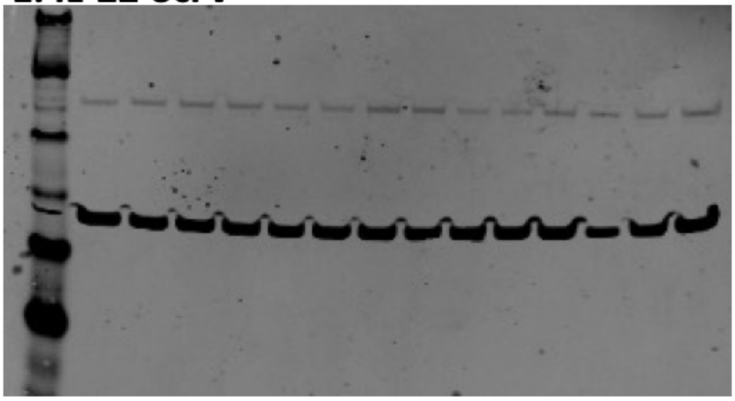

- Top-pSTAT3
- Bottom-β-actin
- Lane 1-2: PBS
- Lane 3-4: IL-22-ScFv-15mins
- Lane 5-6: IL-22-ScFv-30mins
- Lane 7-8: IL-22-ScFv-2hrs
- Lane 9-10: IL-22-ScFv-4hrs
- Lane 11-12: IL-22-ScFv-8hrs
- Lane 13-14: IL-22-ScFv-24hrs

2. IL-22-ScFv-v2

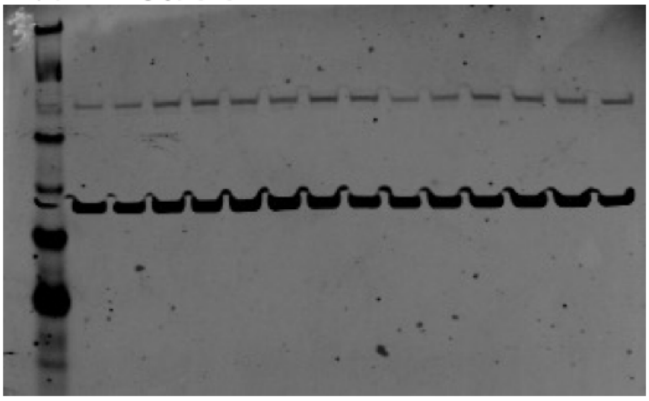

- Top-pSTAT3
- Bottom-β-actin
- Lane 1-2: PBS
- Lane 3-4: IL-22-ScFv-v2 -15mins
- Lane 5-6: IL-22-ScFv-v2 -30mins
- Lane 7-8: IL-22-ScFv-v2 -2hrs
- Lane 9-10: IL-22-ScFv-v2 -4hrs
- Lane 11-12: IL-22-ScFv-v2 -8hrs
- Lane 13-14: IL-22-ScFv-v2 -24hrs

3. IL-22-ScFv-v3

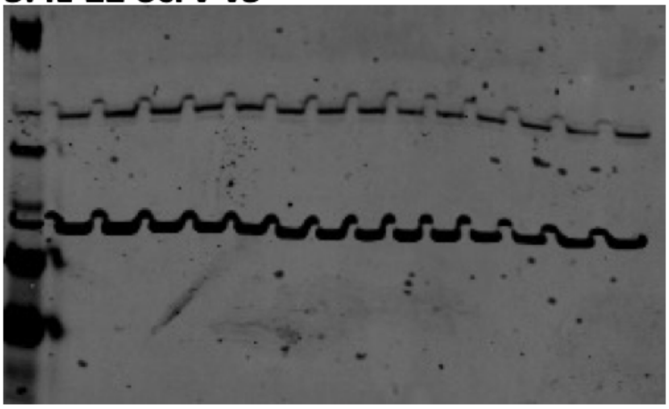

- Top-pSTAT3
- Bottom-β-actin
- Lane 1-2: PBS
- Lane 3-4: IL-22-ScFv-v3 -15mins
- Lane 5-6: IL-22-ScFv-v3 -30mins
- Lane 7-8: IL-22-ScFv-v3 -2hrs
- Lane 9-10: IL-22-ScFv-v3 -4hrs
- Lane 11-12: IL-22-ScFv-v3 -8hrs
- Lane 13-14: IL-22-ScFv-v3 -24hrs

Uncropped western blots – Supplementary Fig. 12d
