# Supplementary material for: Liver and pancreatic-targeted interleukin-22 as a therapeutic for metabolic dysfunction-associated steatohepatitis
Source: Nat Commun. 2024 May 29;15:4528. doi: 10.1038/s41467-024-48317-x (PMC11137118; doi:10.1038/s41467-024-48317-x)
Supplement: Supplementary file 3 — Reporting Summary [file 41467_2024_48317_MOESM3_ESM.pdf]

nature portfolio

Corresponding author(s): A/Prof Sumaira Zia Hasnain

Last updated by author(s): Apr 14, 2024

Reporting Summary

Nature Portfolio wishes to improve the reproducibility of the work that we publish. This form provides structure for consistency and transparency in reporting. For further information on Nature Portfolio policies, see our Editorial Policies and the Editorial Policy Checklist.

Statistics

For all statistical analyses, confirm that the following items are present in the figure legend, table legend, main text, or Methods section.

n/a	Confirmed	
☐	☒	The exact sample size (*n*) for each experimental group/condition, given as a discrete number and unit of measurement
☐	☒	A statement on whether measurements were taken from distinct samples or whether the same sample was measured repeatedly
☐	☒	The statistical test(s) used AND whether they are one- or two-sided<br>*Only common tests should be described solely by name; describe more complex techniques in the Methods section.*
☐	☒	A description of all covariates tested
☐	☒	A description of any assumptions or corrections, such as tests of normality and adjustment for multiple comparisons
☐	☒	A full description of the statistical parameters including central tendency (e.g. means) or other basic estimates (e.g. regression coefficient) AND variation (e.g. standard deviation) or associated estimates of uncertainty (e.g. confidence intervals)
☐	☒	For null hypothesis testing, the test statistic (e.g. *F*, *t*, *r*) with confidence intervals, effect sizes, degrees of freedom and *P* value noted<br>*Give P values as exact values whenever suitable.*
☒	☐	For Bayesian analysis, information on the choice of priors and Markov chain Monte Carlo settings
☒	☐	For hierarchical and complex designs, identification of the appropriate level for tests and full reporting of outcomes
☒	☐	Estimates of effect sizes (e.g. Cohen's *d*, Pearson's *r*), indicating how they were calculated

Our web collection on statistics for biologists contains articles on many of the points above.

Software and code

Policy information about availability of computer code

Data collection	Western blots were imaged on the LI-COR Odyssey CLx system and data was collected using the Image Studio V 5.2 software; Immuno-flourescence microscopy was performed on the Olympus FV3000 Confocal Laser Scanning Microscope and data was collected using the FV31S-SW Viewer Software V2.6; qRT-PCR data was collected using the QuantStudio Real-Time PCR Software V1.7.2; PHERAstar Plus V5.70 (Plate Reader)
Data analysis	Thermo Scientific Proteome Discoverer 3.0 software (Proteomics), MARS Data Analysis Software V4.01 R2 (Plate Reader), Graphpad Prism V10.2.2, ImageJ V1.54i, Visiopharm V2018.4

For manuscripts utilizing custom algorithms or software that are central to the research but not yet described in published literature, software must be made available to editors and reviewers. We strongly encourage code deposition in a community repository (e.g. GitHub). See the Nature Portfolio guidelines for submitting code & software for further information.

Data

Policy information about availability of data

All manuscripts must include a data availability statement. This statement should provide the following information, where applicable:

- Accession codes, unique identifiers, or web links for publicly available datasets
- A description of any restrictions on data availability
- For clinical datasets or third party data, please ensure that the statement adheres to our policy

The proteomics dataset generated and analysed during the current study is available in the ProteomeXchange repository, with the following entry ID: PXD051262.

All other data generated in this study are provided in the Supplementary Information/Source Data file. Source data are provided with this paper.

Research involving human participants, their data, or biological material

Policy information about studies with human participants or human data. See also policy information about sex, gender (identity/presentation), and sexual orientation and race, ethnicity and racism.

Reporting on sex and gender	Pancreatic islets were collected from 2 male and 2 female organ donors
Reporting on race, ethnicity, or other socially relevant groupings	N/A
Population characteristics	The demographic profile of our pancreatic islet donor cohort encompasses a balanced representation of sexes (2 males and 2 females), with ages spanning from 57 to 65 years. The body mass index (BMI) of the donors varies from 26 to 35, placing individuals in the overweight to obese categories, relevant to the study of metabolic tissues. The majority of islet donors do not have a substantive medical history, providing a cleaner analysis of islet function. However, one individual stands out with a history of hypertension and tobacco use.
Recruitment	All samples were obtained from deceased organ donors via the Tom Mandel Islet Transplant Program in Australia
Ethics oversight	The human islet procurement and experimental protocols in this study received approval from the Mater Health Services Human Research Ethics Committee, which provided the ethical oversight.

Note that full information on the approval of the study protocol must also be provided in the manuscript.

Field-specific reporting

Please select the one below that is the best fit for your research. If you are not sure, read the appropriate sections before making your selection.

☒ Life sciences ☐ Behavioural & social sciences ☐ Ecological, evolutionary & environmental sciences

For a reference copy of the document with all sections, see nature.com/documents/nr-reporting-summary-flat.pdf

Life sciences study design

All studies must disclose on these points even when the disclosure is negative.

Sample size	Power calculation conducted by our biostatistician, based on previous data, showed that studies required a sample size of n = 4-12 to achieve a power of 0.8 with an alpha value of 0.05 to detect a true difference in means between the treatment group and control group of 20%.
Data exclusions	Data was excluded based on technical error eg. high background on staining, unacceptable qRT-PCR melt curves
Replication	All experiments were repeated, all attempts at replication were successful
Randomization	All cells/animals in this experiment were randomly allocated to a treatment group.
Blinding	Investigators were blinded to group allocation during data analysis.

Reporting for specific materials, systems and methods

We require information from authors about some types of materials, experimental systems and methods used in many studies. Here, indicate whether each material, system or method listed is relevant to your study. If you are not sure if a list item applies to your research, read the appropriate section before selecting a response.

Materials & experimental systems

n/a	Involved in the study	
☐	☒	Antibodies
☐	☒	Eukaryotic cell lines
☒	☐	Palaeontology and archaeology
☐	☒	Animals and other organisms
☒	☐	Clinical data
☒	☐	Dual use research of concern
☒	☐	Plants

Methods

n/a	Involved in the study	
☒	☐	ChIP-seq
☒	☐	Flow cytometry
☒	☐	MRI-based neuroimaging

Antibodies

Antibodies used	Ki67 (MA5-14520, ThermoFisher Scientific)<br>Phospho-Stat3 (9145, Cell Signaling Technology)<br>Insulin (PA1-26938, ThermoFisher Scientific)<br>Proinsulin (MAB-13361, R&D)<br>Reelin (AF3820, R&D)<br>GFAP (LS-B4775, LifeSpan Bioscience)<br>Alexa Fluor 488 Chicken anti-rabbit IgG (1:500; A-21441, Invitrogen)<br>Alexa Fluor 488 Goat anti-guinea pig IgG (1:1000; A-11073, Invitrogen)<br>Alexa Fluor 555 Goat anti-rabbit IgG (1:1000; A-32732, Invitrogen)<br>Alexa Fluor 555 Goat anti-chicken IgG (1:500; A-21437, Invitrogen)<br>Alexa Fluor 647 Goat anti-mouse IgG (1:500; A-21235, Invitrogen).
Validation	All antibodies used in this study are commercially available and have been validated by the manufacturer and/or other investigators as indicated on the websites:<br>Ki67 - Verified by cell treatment and knockout https://www.thermofisher.com/antibody/product/Ki-67-Antibody-clone-SP6-Recombinant-Monoclonal/MA5-14520<br>Phospho-Stat3 - Published PMID: 37581939, https://www.cellsignal.com/products/primary-antibodies/phospho-stat3-tyr705-d3a7-xp-rabbit-mab/9145<br>Insulin - Published PMID: 35133983, https://www.thermofisher.com/antibody/product/Insulin-Antibody-Polyclonal/PA1-26938<br>Proinsulin - Published PMID: 32644973, https://www.rndsystems.com/products/human-mouse-proinsulin-antibody-253627_mab13361<br>Reelin - Published PMID: 34343491, https://www.rndsystems.com/products/mouse-reelin-antibody_af3820<br>GFAP, https://www.lsbio.com/antibodies/ihc-plus-gfap-antibody-icc-ihc-wb-western-ls-b4775/124468<br>Alexa Fluor 488 Chicken anti-rabbit IgG (1:500; A-21441, Invitrogen); https://www.thermofisher.com/antibody/product/Chicken-anti-Rabbit-IgG-H-L-Cross-Adsorbed-Secondary-Antibody-Polyclonal/A-21441<br>Alexa Fluor 488 Goat anti-guinea pig IgG (1:1000; A-11073, Invitrogen); https://www.thermofisher.com/antibody/product/Goat-anti-Guinea-Pig-IgG-H-L-Highly-Cross-Adsorbed-Secondary-Antibody-Polyclonal/A-11073<br>Alexa Fluor 555 Goat anti-rabbit IgG (1:1000; A-32732, Invitrogen); https://www.thermofisher.com/antibody/product/Goat-anti-Rabbit-IgG-H-L-Highly-Cross-Adsorbed-Secondary-Antibody-Polyclonal/A32732<br>Alexa Fluor 555 Goat anti-chicken IgG (1:500; A-21437, Invitrogen); https://www.thermofisher.com/antibody/product/Goat-anti-Chicken-IgY-H-L-Secondary-Antibody-Polyclonal/A-21437<br>Alexa Fluor 647 Goat anti-mouse IgG (1:500; A-21235, Invitrogen);https://www.thermofisher.com/antibody/product/Goat-anti-Mouse-IgG-H-L-Cross-Adsorbed-Secondary-Antibody-Polyclonal/A-21235

Eukaryotic cell lines

Policy information about cell lines and Sex and Gender in Research

Cell line source(s)	LX2 cells - Gifted from Professor Grant Ramm<br>HepG2 Cells - ATCC<br>MIN6N8 cells - Gift from Professor Josephine Forbes
Authentication	Cell lines were authenticated by morphology check under the microscope prior to each experiment.
Mycoplasma contamination	All cell lines tested negative for mycoplasma contamination
Commonly misidentified lines<br>(See ICLAC register)	No commonly misidentified cell lines were used.

Animals and other research organisms

Policy information about studies involving animals; ARRIVE guidelines recommended for reporting animal research, and Sex and Gender in Research

Laboratory animals	C57BL/6J male mice, 6-8 weeks old at start of experiments. IL-22ra1 fl/fl x Ins2-cre male animals, 8 weeks old at start of experiments. IL-22ra1 fl/fl x Alb-cre male animals, 8 weeks old at start of experiments
Wild animals	This study did not involve wild animals
Reporting on sex	All animals used in this study were male (C57/BL6J background). Female mice of this background are resistant to the development of metabolic dysfunction, and do not gain as much weight when on a high-fat diet.
Field-collected samples	This study did not involve samples collected from the field
Ethics oversight	All experiments were approved by the University of Queensland Animal Ethics Committee (Ethics #2021/AE000426, AE519/16) and conducted in accordance with guidelines set out by the National Health and Medical Research Council of Australia.

Note that full information on the approval of the study protocol must also be provided in the manuscript.

Plants

Seed stocks	N/A
Novel plant genotypes	N/A
Authentication	N/A
